# Quantitative assessment of the erosion and deposition effects of landslide-dam outburst flood, Eastern Himalaya

**DOI:** 10.1038/s41598-024-57894-2

**Published:** 2024-03-25

**Authors:** Xiaolu Dong, Xianyan Wang, Long Yang, Zhijun Zhao, Ronald Van Balen, Xiaodong Miao, Tao Liu, Jef Vandenberghe, Baotian Pan, Martin Gibling, Huayu Lu

**Affiliations:** 1 School of Geography and Ocean Science and Frontiers Science Center for Critical Earth Material Cycling, Nanjing University, Nanjing, 210023 China; 2 College of Geography Science, Nanjing Normal University, Nanjing, 210023 China; 3 Department of Earth Sciences, VU University Amsterdam, 1081 HV Amsterdam, The Netherlands; 4 Henan Key Laboratory of Earth System Observation and Modeling, School of Geography and Environmental Science, Henan University, Kaifeng, 475004 Henan China; 5 Department of Hydrology and Atmospheric Sciences/Department of Geosciences, University of Arizona, Tucson, AZ 85721-0011 USA; 6 College of Earth and Environmental Sciences, Lanzhou University, Lanzhou, 730000 China; 7 Department of Earth and Environmental Sciences, Dalhousie University, Halifax, NS B3H 4R2 Canada

**Keywords:** Geomorphology, Hydrology, Natural hazards

## Abstract

Both regular flow and infrequent outburst floods shape the mountain landscape, but their relative contributions have been widely debated, in part due to the paucity of quantitative data on historical outburst floods. In June 2000, an outburst flood was triggered by a landslide-dam failure in a rapidly exhumed region of the Eastern Himalaya. To investigate the role of this kind outburst flood on landscape evolution, we employ topographic differencing, satellite imagery, and 2D hydraulic simulations to quantify the equivalent erosion and deposition within ~ 80 km flood route downstream of the breach. The flood lasted for ~ 10 h, with a peak discharge of 10^5^ m^3^/s, leading to average erosion of 10 m, and contributed ~ 1–2 × 10^3^ times more sediment than over long-term mean fluvial processes. The flood produced extensive lateral erosion, which triggered a threefold widening of the valley floor and abundant subsequent landslides. The ubiquitous boulder bars deposited in the channel inhibited incision, and facilitated lateral erosion after the flood. The resulting channel configuration and extensive bank erosion continue to affect fluvial dynamics until the next catastrophic flood that remobilizes the boulders. Our quantitative findings highlight the profound importance of recurrent outburst floods for gorge development and landscape evolution in Eastern Himalaya.

## Introduction

Rivers are the most important surface agents that shape landscapes through erosion and sediment transport, and understanding how they accomplish their work is a key issue in geomorphology^1,2^. Outburst floods may play a crucial role in sculpting deeply incised fluvial canyons that are generally blocked by landslides or glaciers^3–10^. However, the exact impact of such catastrophic events on long-term landscape evolution remains uncertain because historical outburst floods are rare, short-lived, and hazardous, making them difficult to document, especially in steep mountainous areas.

**Figure 1:**
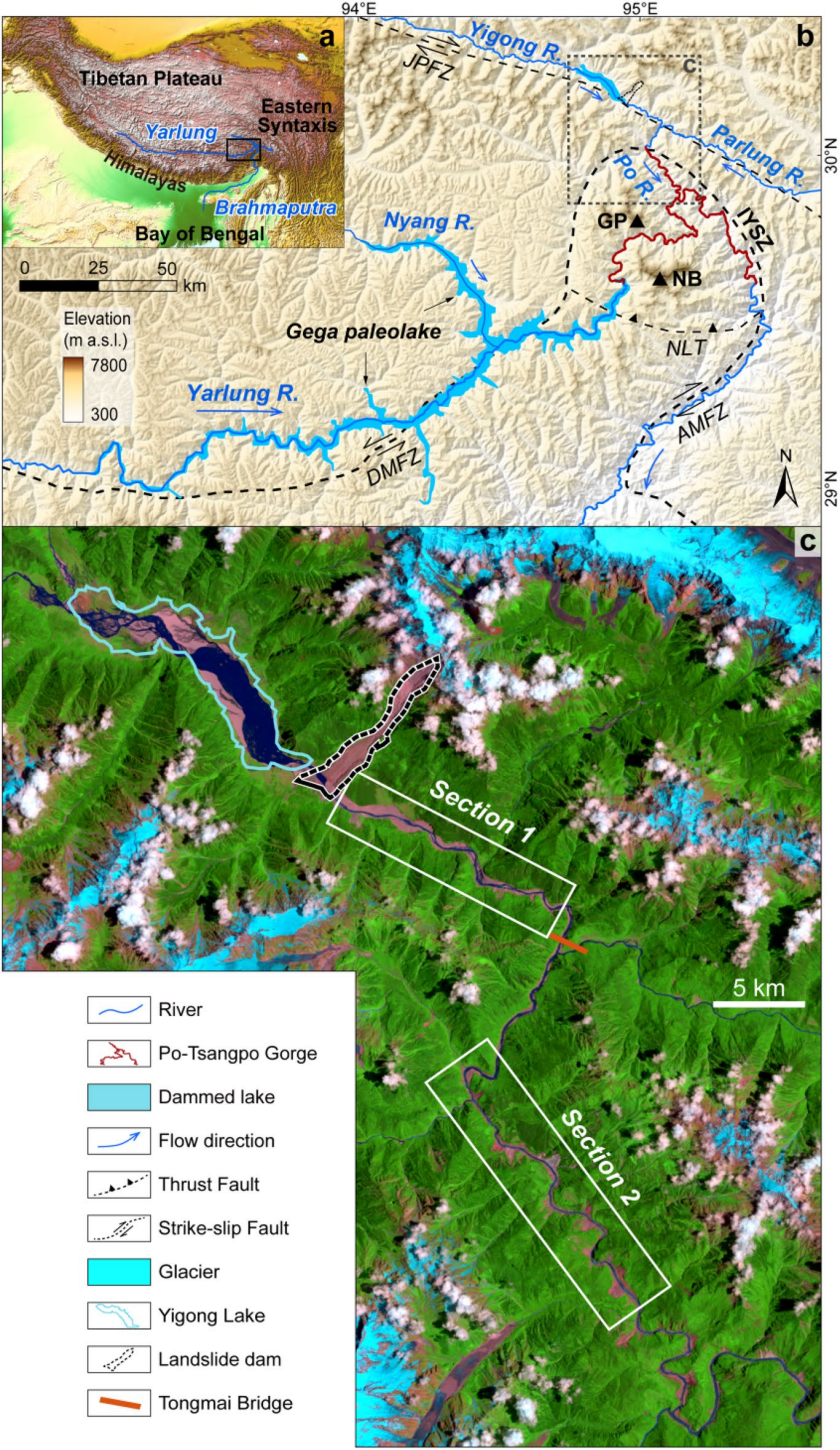
The regional background of the Yigong Basin in which the outburst flood occurred. (**a**) Major fluvial drainage of the eastern Himalayan region. (**b**) Drainage patterns, geomorphic and tectonic background^14,33^ for areas surrounding the Tsangpo Gorge. The Gega paleolake, reconstructed from Montgomery et al.^22^, had a stored water volume of 81 km^3^ with peak discharge of 1–3 × 10^6^ m^3^/s^22,25^. NB: Namche Barwa peak (7782 m.a.s.l.). GP: Gyala Peri peak (7294 m.a.s.l.). DMFZ: Dongjiu-Milin fault zone. AMFZ: Aniqiao-Motuo fault zone. JPFZ: Jiali-Parlung fault zone. NLT: Namu-La thrust. IYSZ: Indus Yarlung suture zone. (**c**) Landsat-5 imagery on July 24, 2003 shows the source of the 2000 Yigong outburst flood as well as its landslide-dam and pathway. The map was created using a licensed ArcGIS 10.2 software (https://support.esri.com/zh-cn/overview).

**Figure 2:**
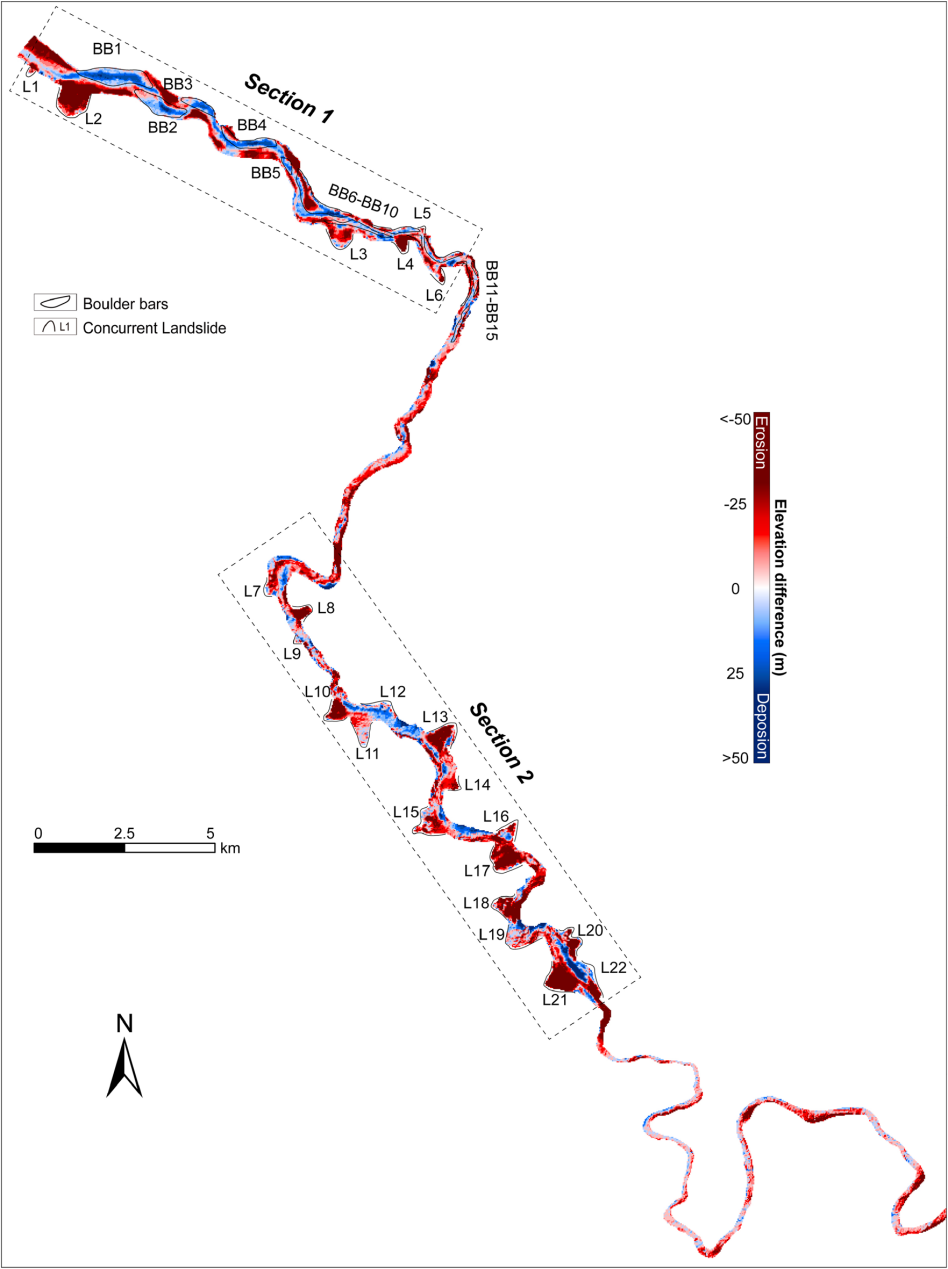
Changes in topography based on pre-flood and post-flood digital elevation models (DOD-derived), showing patterns of erosion and aggradation along the ~ 80 km flood route downstream of the landslide-dam. Areas with positive DOD values and sediment aggradation represent newly formed boulder bars on both sides of the river, whereas areas with negative DOD values represent bank erosion, channel incision, and landslide erosion (L1–L22). The detailed DOD results of boulder bars and concurrent landslides are shown in Supplementary Fig. 7.

The Yigong outburst flood on the Himalayan in June 2000, with well-preserved geomorphic and sedimentary evidence, provides a rare opportunity to quantitatively assess the geomorphic impacts of such an extraordinary event (Fig. 1). Turzewski et al.^11^ have identified sustained high bed shear stress, bedrock incision, sediment transport, and deposition during the Yigong superflood (a peak discharge of 10^5^ m^3^/s). However, their study was limited to a two-dimension perspective and lacked the quantitative exploration of geomorphic changes. In this study, we aim to expand on their findings by using direct observations to quantify erosion and deposition during the outburst flood. Our approach involves incorporating a 3D perspective to better understand the rapid evolution of the earth surface in response to such dynamic flooding event in a rugged mountainous landscape. To evaluate spatial patterns of erosion and deposition, we utilized topographic changes along the ~ 80 km flood path downstream of the collapse site based on pre-flood and post-flood digital elevation models (“DOD” hereafter) (Fig. 2). Additionally, we employed a 2D hydraulic simulation to reconstruct flood discharge and shear stress, especially for sites where subsequent landslides occurred or boulder bars accumulated. By combining hydraulic modeling with observations of topographic changes before and after the event, we were able to assess the geomorphological processes associated with this sudden outburst flood affecting steep channels, valleys and hillslopes. Finally, we compared the erosional effects of this flood with long-term (10^3^–10^6^ years) denudation rates in the Tsangpo Gorge region derived from cosmogenic nuclide analysis and thermochronology^12–14^ in order to determine its relative contribution to long-term denudation.

## Regional setting and the 2000 extreme Yigong outburst flood event

We document the geomorphic effects of an outburst flood in the Namche Barwa-Gyala Peri (NBGP) massif of the Eastern Himalayan Syntaxis, which is among the most actively uplifting parts of the mountain belt and has undergone rapid exhumation (> 5 mm/y) during the late Cenozoic^12–18^. The Yarlung River cuts through this syntaxis between two peaks with elevations exceeding 7 km, forming a narrow bedrock gorge ~ 5 km deep, making it one of Earth’s deepest gorges (Fig. 1a)^12–18^. This Yarlung Tsangpo Gorge descends ~ 2 km over a distance of ~ 150 km (average gradient of 0.013) cutting into fragile bedrock characterized by well-developed joint fractures (see Supplementary Text). Abundant moisture from the Indian Ocean moves along the Yarlung River valley and enters the Tsangpo Gorge to nourish glaciers on both peaks^19^. The strong tectonic activity, high topographic relief, and glaciers creates conditions for intense surface processes including landslides, glacier avalanches, damming and subsequent outburst floods^20–24^. Previous studies in this river system have documented dam breaches during late Pleistocene and Holocene that resulted in superfloods and even megafloods (reaching peak discharge up to 10^6^ m^3^/s) (Fig. 1b), based on sedimentary and geomorphic archives^20–23,25–30^. With their immense stream power, these outburst floods have played a crucial role in long-term morphological evolution of the gorge^20,21,23,25,29^, alongside high-frequency meteorologic floods. However, limited direct observations exist to constrain erosion and deposition from such outburst floods.

In April 2000, the Yigong River, a major tributary of the Yarlung River (Fig. 1b), was blocked by one of the largest mass failures worldwide^31^ since 1900. The landslide was triggered by excessive meltwater and rainfall and impounded approximately 2 × 10^9^ m^3^ of water in Yigong Lake (Fig. 1c) before the dam was breached on 10 June 2000, two months after the impoundment^31^. The subsequent rapid drainage of the lake led to a cataclysmic outburst flood that descended through the Tsangpo Gorge towards the Himalayan range front (Fig. 1b). The peak discharge at Tongmai Bridge, ~ 17 km downstream of the landslide-dam (Fig. 1c), reached ~ 12 × 10^4^ m^3^/s, ~ 36 times greater than the normal meteorologic river flooding of this river^32^. This particular outburst flood stands as the world’s second-largest recorded landslide-dam outburst flood^31^, which resulted in 37 landslides and extensive kilometers-scale boulders bars^11^.

## Materials and methods

### Mapping of topographic changes

The Shuttle Radar Topography Mission (SRTM), conducted aboard the space shuttle Endeavour in February 2000^34,35^, provides the pre-flood topographic data with a spatial resolution of 1 arc-second (https://earthexplorer.usgs.gov/). On the other hand, The Advanced Land Observing Satellite (ALOS) data, acquired after its launch in January 2006^34,35^, offers the post-flood topographic information at the same spatial resolution (https://search.asf.alaska.edu/). To determine the topographic changes resulting from the 2000 Yigong outburst flood, the ALOS post-flood dataset was subtracted from the SRTM pre-flood topography dataset. This operation yields a difference map that represents the change in elevation, referred to as the Digital Elevation Model of Difference (DEM of Difference, orDOD). This DOD map allow for the visualization and analysis of the geomorphic impact of the flood event.

Prior to calculation and interpretion the elevation difference, the vertical accuracy of the two different data products was compared^36^. To ensure consistency on the same horizontal datum plane, we utilized official elevations from three international airports (Qamdo Bangda Airport, Gongga Airport, and Nyinchi Airport, https://www.fsaerodata.com/) around Yigong Lake as control points for calibrating the elevation data (Supplementary Table 1). In addition, the valley floor and hillslope areas upstream of the Yigong flood, which thus were unaffected by the outburst flood, have been instrumental in demonstrating the calibration of the horizontal datum planes of these DEMs (Supplementary Fig. 3a and b). The results indicate that after calibration with an error range of ~ 5 m^37^, both DEMs exhibit satisfactory vertical precision (Supplementary Fig. 3). A positive value of DOD indicates accumulation while a negative value signifies erosion caused by the flood (Fig. 2). We assumed that, compared with effects induced by the 2000 flood, topographic changes were negligible between February 2000 and 10 June 2000 (the date of the outburst flood) and between 10 June 2000 and the acquisition time of ALOS data in 2006, considering that there is no catastrophic surface process or earthquake during this period. We further assessed reliability and verification of DOD results along with uncertainties (see Supplementary Text). We utilized the topographic differencing map, validated by Landsat and Google Earth images, along with a field expedition, to delineate topographic changes and outline the erosional and depositional volumes by the 2000 Yigong outburst flood. In order to quantify the mean erosion depth resulting from this outburst flood that extensively scoured and destabilized both valleys and hillslopes, we divided the volume by the corresponding geomorphic change area. Subsequently, we contextualized this mean erosion depth within a long-term perspective specific to the local massif (see Supplementary Text).

### Flood hydraulics

The HEC-RAS software (Hydrologic Engineering Center’s River Analysis System)^38^, was utilized to model the dam break simulation for obtaining the breach outflow and outburst flood hydrodynamic routing with 2-D shallow water equations. This software has been successfully applied in modeling potential dammed lake outburst flood events in steep terrains like the Himalaya^39–41^, including the Yigong outburst flood^42,43^. However, previous studies lacked coupling the user-friendly HEC-RAS model between the dam-break simulation and 2D hydraulic simulation, which could be an effective method for reconstructing different dynamic evolutions assuming different dam-break processes given that few paleoflood deposits but dammed-lake deposits have been well preserved. Here, we simulate the 2000 Yigong outburst flood again assuming a stable riverbed during the flood to explore an alternative method for reconstructing outburst floods through the Yarlung region.

The simplified dam-break flood process is reconstructed based on the SRTM DEM prior to the event, consisting of a storage area (Yigong Lake), a landslide dam, and a 2D flow area in the HEC-RAS model. (1) Reservoir. The location of Yigong Lake (Fig. 1) was determined using remote sensing images from May 12, 2000. The storage capacity curve of Yigong Lake (Supplementary Fig. 11d) was inputted, and the initial elevation of the storage area is inferred to be at 2265 m asl according to Delaney and Evans^31^. (2) Dam break simulation. The HEC-RAS dam break model utilizes a parametric model to predict the final break parameters and then applies a simplified physical model to solve for broad-crested weir outflow. The unconsolidated Yigong landslide dam failed due to overtopping, assuming instantaneous dam failure at the breach site. The physical shape parameters of the landslide-dam before and after the flood were used based on an inferred height (~ 55 m) of the landslide-dam by Delaney and Evans^31^, in order to calculate a discharge hydrograph at the dam breach. Detailed parameters of the dam break model can be found in Supplementary text and Supplementary Table 3. (3) Hydrodynamic routing simulation. The simulations are configured with a nominal mesh resolution of 30 m using a time step of 1 s over a simulation time period lasting for 17 h. Previous studies^44,45^ have shown low sensitivity regarding the choice of Manning coefficient (n) which is used for calculating very large discharges in narrow deep bedrock canyons. The coefficient is thus assigned uniformly as 0.04 for the whole domain. The impact of the background flow on flood depth can be disregarded since discharge from the outburst flood was at least 100 times larger (see Supplementary text). Therefore, the inflow discharge into the lake is set to 0. Moreover, the breach hydrograph obtained through the dam break model and the normal depth were considered as the upstream and downstream boundary conditions, respectively, during dynamic flood routing. The HEC-RAS simulations provide hydraulic parameters such as flow depth, velocity, shear stress and discharge for each cross-section.

Due to the absence of real-time monitoring, it is challenging to distinguish and quantify bedrock and alluvial sediments eroded and excavated by the outburst flood. However, based on the continuous presence of high shear stress (e.g., > 1 kPa) during the flood and the observed post-flood landforms, we tentatively assume that areas displaying freshly eroded bedrock and alluvium imply erosion of both types of material.

### Concurrent landslide mapping and volume estimation

#### Landslide mapping

Landslide mapping over a distance of ~ 80 km downstream from the breach between 1990 and 2020 was conducted using Landsat remote sensing images, primarily obtained during relatively clear sky conditions from April to September. This period is characterized by robust vegetation growth and weak mountain shadow, which enhances image accuracy and reliability compared to winter conditions for ground features recognition and classification. Supplementary Table 2 shows the selection of Landsat data. Field validation of landslide inventories, location and size determination, was performed along a ~ 30 km stretch downstream from the landslide-dam where roads were accessible.

The following steps were employed to identify and map concurrent landslides^46^: (1) Supervised classification of features using ENVI software was used to extract landslides connected to the valley floor. (2) Remote sensing images show similarities in areas with landslides, floodplains, and roads. Independent research has shown that areas with slopes < 20° have very low landslide densities^47^, so results were re-extracted for areas with slopes > 20°. (3) Finally, visual interpretation confirmed or modify the landslide boundaries.

#### Landslide volume estimation

The landslides were identified and numbered as L1–L22 (Fig. 2). The height/erosion depth of each landslide was calculated using the DOD value was used to calculate. The total volume of a landslide body is the sum of the DOD values of all grid cells multiplied by the unit grid cell area^48,49^. Thus, we calculated the volume of each landslide individually as:

1 $$\begin{array}{*{20}c} {V_{Lj} = \mathop \sum \limits_{i = 1}^{n} H_{i} \times A_{cell} } \\ \end{array}$$

where ${V}_{Lj}$ is the volume of the landslide numbered $Lj$, with $j$ = 1–22; $n$ is the total number of grid cells for that particular landslide; ${H}_{i}$ is the elevation difference of each grid cell in the landslide area, that is, the DOD value of each grid cell; ${A}_{cell}$ represents the area of the unit grid cell (i.e., 30 m × 30 m). However, due to the difference in vertical accuracy between DEM data on hillsides versus valley floors, there may be some error associated with calculating landslide volumes using DOD values alone (see Supplementary Text). Therefore, empirical formulas derived from a dataset comprising over 4,000 bedrock and soil landslides—including those from the Namche Barwa massif^24,50^—were used to verify our calculations regarding landslide area-volume relationships (Supplementary Fig. 7d).

### Valley floor width mapping

We manually delineated the regional valley floor outline by analyzing Google Earth images from 1999 to 2006 and Landsat remote sensing data from 1999 (Cook et al., 2014)^51^. For each image, we carefully traced the boundaries of the valley bottom, which is characterized by a distinct change in slope at the upper edge of the steep walls of the gorge, often indicated by sparse vegetation cover as Cook et al.^51^. Then we delineated the outlines of the valley floor based on the valley floor edge, which was divided into 53 segments with an average length of 1.5 km based on 80-km channel centerline length. The total area and channel centerline length for 53 parts along the flood-route have been manually measured. The ratio between the total area of each segment and its corresponding channel centerline length before and after the flood represents the specific valley floor width, thus reducing the subjective judgment of directly measuring the valley floor width.

## Results

### Spatial patterns of erosion and deposition

**Figure 3:**
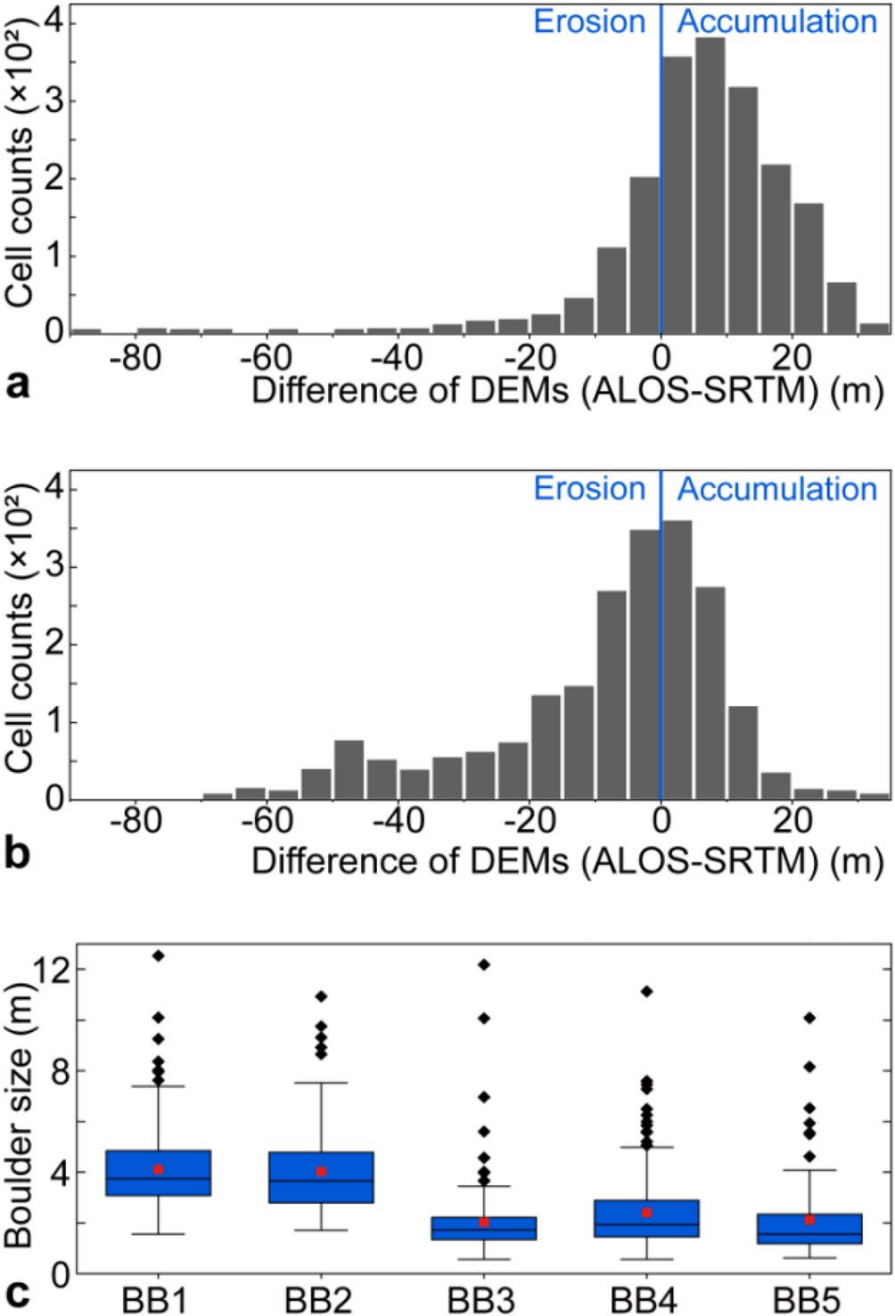
(**a**, **b**) Patterns of erosion and aggradation revealed by the distribution of DOD values at the location of the pre-flood (**a**) and post-flood channel (**b**) showing erosion and deposition by the flood. The blue line marks the transition between erosion and accumulation revealing channel migration. (**c**) Size of boulders in the bars, based on images from unmanned aerial vehicles (UAV, 0.14 m resolution, taken in August 2019) and field measurements. The blue bars indicate the boulder bars accumulated by the flood. The box spans the interquartile range, the red dot denotes the average, the line denotes the median, 25th to 75th percentiles and whiskers denote Q3 ± IQR (outlier truncation point). The locations of BB1–BB5 are shown in Fig. 2.

**Figure 4:**
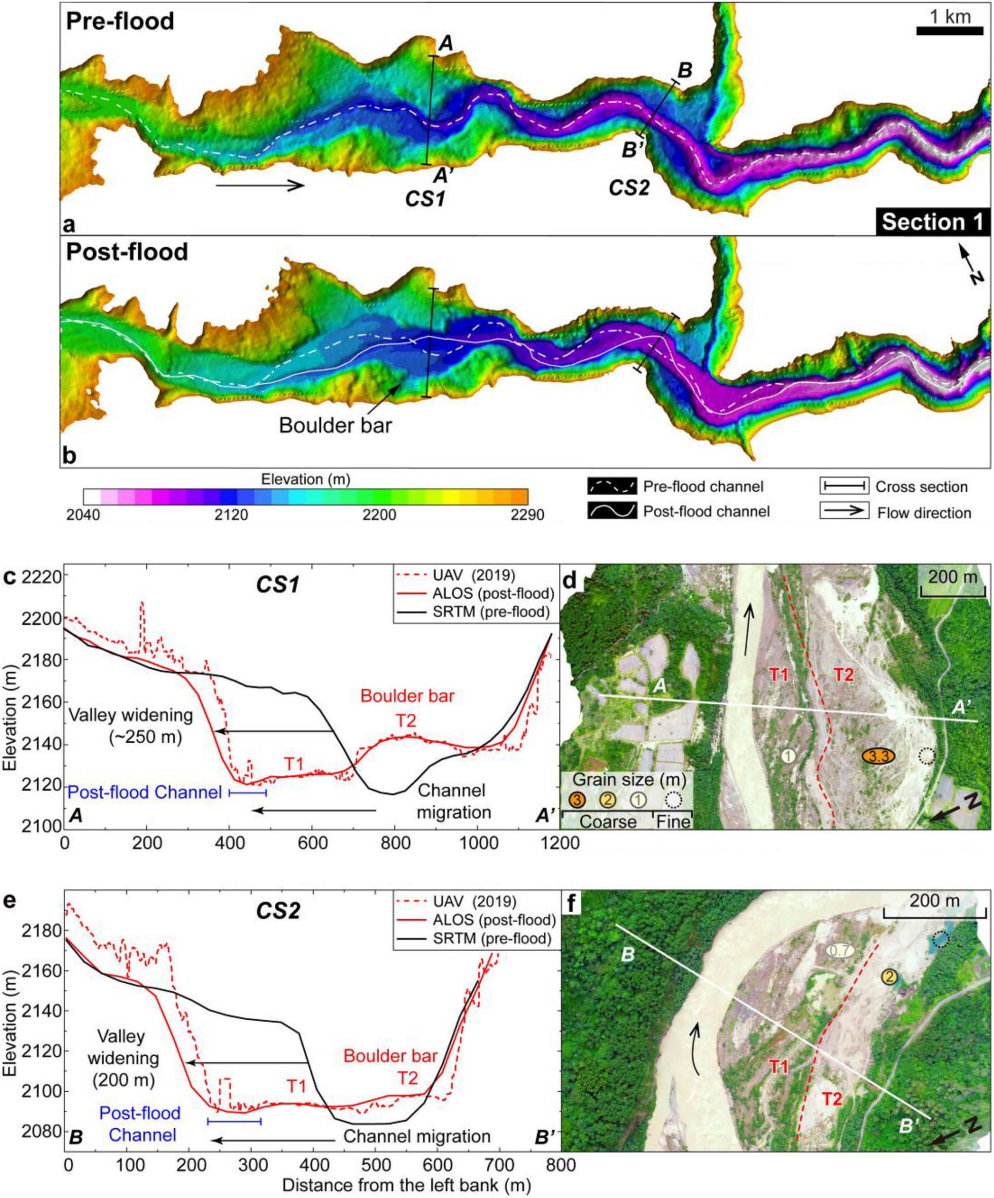
Terrain before and after the flood in the 17 km reach of the Yigong River, from the breach to Tongmai Bridge (Section 1). (**a**) Topography before the flood from the Shuttle Radar Topography Mission (SRTM), ~ 30 m digital elevation model^34^. (**b**) Topography after the flood from the Advanced Land Observing Satellite (ALOS), ~ 30 m digital elevation model^34^. Comparison of the two digital elevation models indicates valley floor widening, channel migration, and deposition of boulder bars. CS1 and CS2 denote the location of cross-sections. (**c**, **e**) Comparison of valley and channel elevations and positions in SRTM (pre-flood), ALOS (post-flood), and UAV data (post-flood) models for CS1 (**c**) and CS2 (**e**). At CS1, two topographic steps (T2 and T1) formed by the flood are ~ 20 and ~ 4 m above the current channel. At CS2, T2 and T1 are ~ 8 and ~ 4 m above the current channel. The T2 at CS2 was damaged, but still left a small amount of boulder for particle size measurement. (**d**, **f**) Unmanned aerial vehicle images of CS1 (**d**) and CS2 (**f**). Dark brown-yellow areas are large blocks (> 1 m diameter), whereas bright brown-yellow areas are fine-grained gravels and sands. The numbers inside the circle represent the average particle size of coarse particles on each platform. The maps were created using a licensed ArcGIS 10.2 software (https://support.esri.com/zh-cn/overview).

Flood-induced erosion and deposition occurred along the valley as a result of channel migration, lateral scour, and the accumulation of large boulder bars (Figs. 2, 3, 4). The absence of vegetation marked the margin of the valley floor, which was determined through mapping Google Earth images (Fig. 4c) and verified in the field. Flood-related erosion cut into both pre-existing alluvium (landslides and debris fans) and the bedrock on the valley side (Supplementary Fig. 2). Approximately 52.6 × 10^6^ m^3^ of sediment and bedrock were eroded by the flood, while ~ 31.2 × 10^6^ m^3^ of sediment were deposited within a downstream reach of 17 km reach to Tongmai Bridge (Section 1; Fig. 2). This means that a net amount of ~ 21.4 × 10^6^ m^3^ of material was eroded in this particular reach.

Prominent changes can be observed in the shape of valley cross-sections (e.g., CS1 and CS2 sections; Fig. 4), where vegetation on both channel banks was destroyed by the flood and sediments were redistributed accordingly (Fig. 4c, e). On the right margin of the valley, gravel with large blocks accumulated on top of two topographic steps related to the flood was denoted as T1 and T2 respectively; notably, particles on T2 higher platform are significantly coarser than those found on the T1 lower platform (Fig. 4c–f). Lateral erosion widened the valley floor by ~ 250 m while causing a shift in channel position towards the left margin by ~ 300 m; during this process, a bend was cut off resulting in straightening of the channel (Fig. 4b). Over a stretch of ~ 10 km downstream from the breach, the channel experienced lateral migration and multiple re-locations, resulting in a reduction of 416 m channel length (Fig. 4a, b). These adjustments reflect how that channel adapted to accommodate the high discharge and generate the required transport capacity for the substantial sediment load carried during the outburst flood. Within the channel-migration reach, half of the current channel banks are flanked by outsized boulder bars on one side and steep valley walls on the other side (Fig. 4 and Supplementary Fig. 1). Therefore, in the long-term geomorphological recovery following this extreme event, lateral erosion from regular flows is expected due to the unmovable boulders and fractured rock in the valley wall.

### Erosion and concurrent landslides

The flood-induced lateral erosion impacted the valley-margin hillslopes in two ways. Firstly, low-slope valley margins experience bank collapse resulting in parallel retreat of the valley sides, leading to a threefold increase in the width of the valley floor from 99 ± 30 to 296 ± 27 m (Fig. 4). This process involved significant erosion and transport of large sediment volumes as well as excavation of bedrock under high shear stress of ~ 4–20 kPa (Supplementary Fig. 5b). Secondly, concurrent landslides destabilized steeper valley margins with slopes between 32° and 49° (Supplementary Fig. 7c), at the threshold of failure^24^. Along ~ 80 km of the flood path below the dam breach, there was a threefold increase in cumulative landslide area after the flood (Fig. 2). Although mass movements are common within this region^24^, this extreme event was remarkable for its numbers/areas of failures and for impacting high-elevation mountainsides. The equivalent mean erosion depth caused by the flood on affected landslides is estimated at 21 ± 10 m^3^/m^2^ (Fig. 2). These landslides affected the valley-margin topography up to about 500 m above the valley floor (Supplementary Fig. 4), supporting that low-frequency and high-magnitude floods can trigger a pulse of landslides from valley walls to considerable elevations beyond just adjacent hillslopes near channel bases^21,24^.

**Figure 5:**
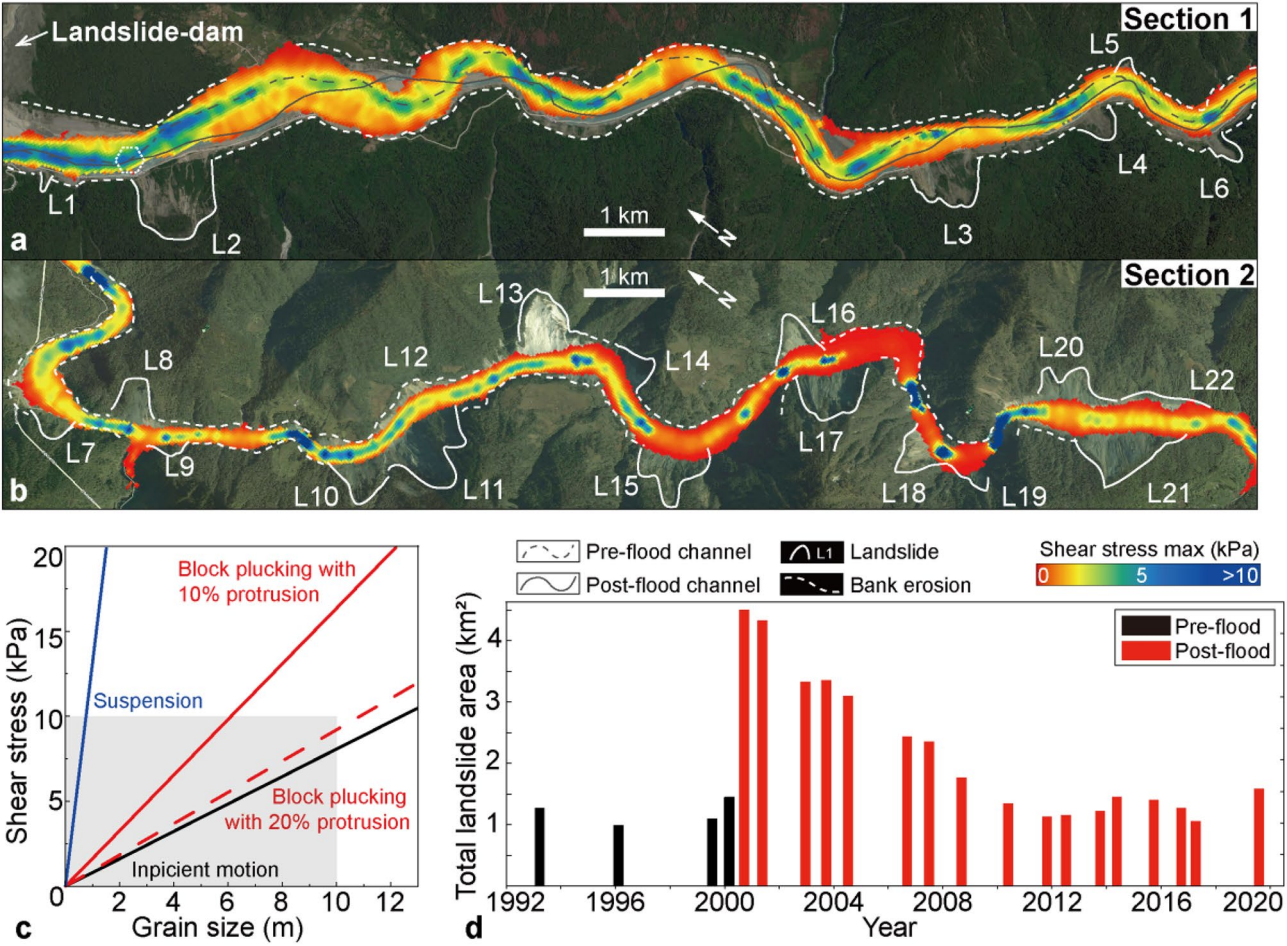
Erosional landforms generated by the flood, and flood hydraulics. (**a**, **b**) Distribution of the maximum shear stress calculated using the HEC-RAS model during the flood, and concurrent landslides, for Section 1 (**a**) and Section 2 (**b**) (see locations in Fig. 1c). (**c**) Thresholds of shear stress for a given block size for various erosion processes through the Tsangpo Gorge, indicating that the outburst flood can entrain or pluck meter-scale blocks. The gray square shadow indicates the main shear stress range of the 2000 Yigong outburst flood. (**d**) Changes in total landslide areas within ~ 80 km downstream of the dam between 1992 and 2020, determined from Landsat images (Supplementary Table 3).

Significant lateral erosion occurred on both sides throughout the entire flood path rather than being confined only to concave banks (Fig. 5). Concurrent landslides were related to either concentrated high shear stress near valley constrictions such as L4 and L8 (Fig. 5) or flood flow shocking hillslopes with high angles from valley walls like L2 and L21 (Fig. 5). For example, the shear stress passing through landslides L20 and L21 is ~ 2–3 k Pa (Fig. 5). At these locations, the flood flow disperses from valley constrictions and rapidly moves towards both sides of the river valley, causing landslide erosion depths reaching up to 10 m (Supplementary Fig. 7). By combining field surveys with simulation results, it is possible to capture the extent of bedrock erosion caused by the powerful outburst flood. In Section 1, where a near-vertical valley side exists at the constriction, bedrock is exposed along the left bank. Here, the high flood shear stress (2–5 kPa; Supplementary Fig. 2) has led to plucking of meter-scale boulders (Fig. 5c), potentially facilitated by vortices or kolks generated by the outburst flood^52^. Furthermore, evidence of bedrock plucking can be observed in broken slate outcrops located ~ 10 m above the modern water surface (Supplementary Fig. 2). In the Tsangpo Gorge, where the flood discharge reached about 5 × 10^4^ m^3^/s—a magnitude rarely achieved by typical meteorological floods^52^—shear stress was sufficiently high (5–20 kPa; Fig. 5c) to pluck blocks measuring between 6 and 12 m in size (Fig. 5c), highlighting the immense erosive capability of this flood event. The combination of extensive erosion potential and landslide damming contributes to significant sediment loads involved in flooding.

### Deposition

**Figure 6:**
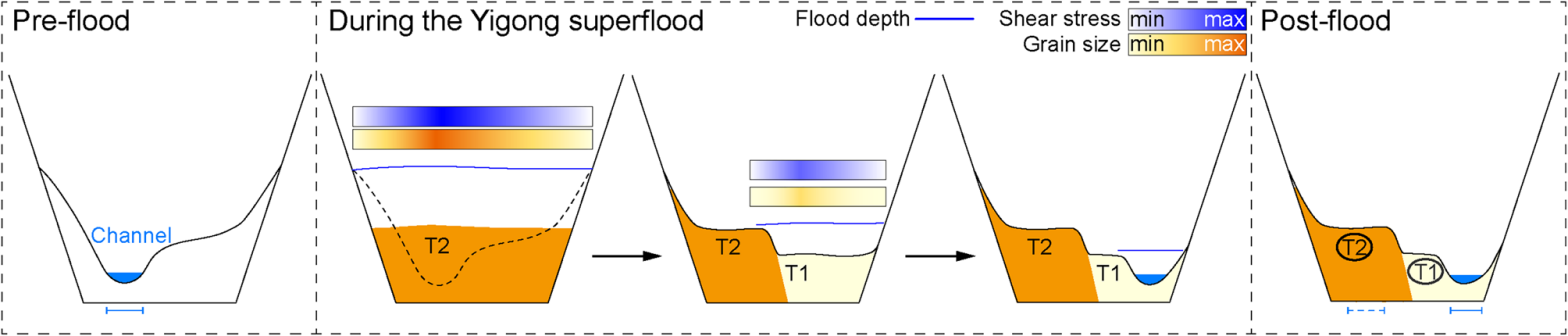
Schematic diagram illustrating flood dynamics and consequent geomorphic change. The valley evolution (valley floor widening, boulder bars deposition) and channel migration were caused by the 2000 Yigong outburst flood.

The immense transport capacity of the flood and the substantial amount of sediments supplied by landslides resulted in the formation of 15 large bars composed of imbricated boulders on both sides of the channel between the landslide-dam and Tongmai Bridge (Fig. 2). Flood-modified sediments accumulated downstream of the landslide-dam to a thickness of ~ 10 ± 5 m (BB1–BB15; Fig. 2), consisting of mixed fine- and coarse-grained deposits containing boulders and blocks with mean diameters ranging from about 2–4 m (BB1-BB5; Fig. 3c). These large blocks exhibit entrainment shear stresses of 1.6–3.2 kPa, indicating significant competence and transport capacity (Fig. 5c). While these blocks are prominent along the pre-flood channel where shear stresses were maximal (Supplementary Fig. 6), they are not concentrated along the post-flood channel, suggesting a complex relationship between boulder bars, flood dynamics and channel migration. Although our simulation does not account for any topographic changes that may have occurred during the course of this flood event, we infer that the landscape evolution throughout the outburst flood proceeded as follows (Fig. 6). At initial breaching (t < 1 h), high shear stress in the pre-flood channel (5 kPa) caused peak discharge to entrain the most coarse outburst flood deposits which were then occupied within existing channel as the discharge waned (Fig. 6). The continued decrease of discharge and shear stress (e.g., to < 1.6 kPa), coupled with increased roughness around boulders, led to the deposition of finer particles on both sides of the pre-flood channel resulting in the accumulation of a platform of higher T2 (see grain size distribution in Fig. 4d, f) (Fig. 6 and Supplementary Fig. 6). Then the recession of the flood and contraction of its flow led to local high shear stress flushing the reach occupied by fine deposits upstream, resulting in lateral channel modification (Fig. 6). Subsequently, the outburst flood formed a lower T1 accumulation platform with the coarser deposit (see grain size distribution in Fig. 4d, f) (Fig. 6). Therefore, with the flood hydraulic dynamics, fine particles and coarse particles on the same side of the river can be observed in cross-sections after the flood (Fig. 4).

Large boulders were not transported during the recession of the flood and would not be transported by following meteorological floods due to their magnitude, but instead promote lateral channel migration and reorganization when occupying boulder bars. It is excepted that transport of these boulders will resume by future high-magnitude outburst floods and deposited as new bars downstream, armoring and roughening the riverbed there.

## Discussion

### Contribution of the outburst flood to denudation in the Eastern Syntaxis

For the 2000 Yigong flood, we utilized the elevation difference map (DOD data) in conjunction with post-flood aerial imagery and field checks to estimate the volume of topographic changes. Our calculations over an 80 km studied reach indicate that ~ 2.6 × 10^8^ (25.8 ± 0.15 × 10^7^) m^3^ of material was eroded in excess of deposited material, with ~ 1.6 × 10^8^ (15.5 ± 0.13 × 10^7^) m^3^ contributed from erosion in the valley floor and ~ 1 × 10^8^ (10.3 ± 0.06 × 10^7^) m^3^ from concurrent landslides (Fig. 2). When normalized by valley floor area, the equivalent average valley erosion depth is 8 ± 5 m^3^/m^2^, while calculated from concurrent landslides’ volume and area, and equivalent landslide erosion depth is 21 ± 10 m^3^/m^2^ (Fig. 2). Integrating these results based on their respective areas yields an equivalent average erosion depth for the study area of 11 ± 5 m^3^/m^2^ (Fig. 2).

Geomorphic models for the NBGP massif suggest a close coupling between river incision, sediment evacuation in the Yarlung Tsangpo gorge, and vertical rock uplift; furthermore, landslide erosion rates along the rivers correlate with exhumation rate and stream power^24,53^. These models imply that denudation in the massif is primarily influenced by incision along the Tsangpo River and its tributaries, which adjust to keep pace with rock uplift. Therefore, as an initial approximation, denudation rates for the NBGP massif can be compared to long-term mean vertical erosion rates and also to the average erosion depth resulting from the 2000 flood. Denudation rates of 5–10 mm/yr are considered appropriate comparators (see Supplementary Text), derived from thermochronological and cosmogenic data^12,14–17,24,53^ that constrain long-term geomorphic rates. We estimate that erosion caused by the 2000 Yigong flood was equivalent to ~ 1–2 k.y. of long-term mean valley erosion. Additionally, our direct topography data suggests that Lang et al.^21^ predictions of flood-induced erosion calculated on a plucking length scale may underestimate its geomorphic impact.

Short-duration extreme events are discrete and perturbs the landscape evolution during long periods of background geomorphological change driven by tectonic uplift and climate^54^. Following this event which effectively performs geomorphic work, the affected landscape enters a period of recovery during which the geomorphological system gradually returns to its background conditions and mean long-term sediment yields^55–57^. Over the past seven millennia, at least nine landslide-dam outburst floods have been recorded at Yigong Lake, including this modern event, with a recurrence interval of ~ 700 years^43,58^, whereas this 10 h extreme event generated significantly more sediment export than the typical long term average exceeding three orders of magnitude. Dammed lakes such as Gega paleolake in the Yarlung Valley (Fig. 1b) were filled and breached repeatedly^21–23,27,28^. These very large lakes (80–800 km^3^) which lasted for a long time (between 10^3^ and10^4^ years) may have released megafloods with unit stream power of 10^4^–10^6^ W/m^2^ and discharges some ten times greater (> 10^6^ m^3^/s) than those observed during the 2000 Yigong flood^21,22,25–27^, exceeding the largest known present-day floods in the study area by an order of magnitude^52^. The denudation of the megaflood (discrete process) was quantified by Borgohain et al.^26^ to be equivalent to the material that would be produced over ~ 8000 years of erosion within the NBGP massif, at a rate of ~ 9 mm/a. They also identified at least 5 megaflooding events since 15 ka, occurring with a frequency of less than 3000 years^26^. Considering the availability of sediment sourced from steep valleys and hillslopes, as well as the occurrence frequency of outburst floods, it is challenging for both the Yigong River and Tsangpo Gorge to recover to background transportation rates. This difficulty is particularly pronounced in the Eastern Himalayan region, which is known for being a hazard hotspot for outburst floods^59^. Consequently, based on recurrent perturbations caused by these floods, it can be concluded that both the Yigong River and the Tsangpo Gorge should be classified as being in an unsteady state in the long term^54^. This further emphasizes how extreme events have had a lasting impact on landscape evolution in the Tsangpo Gorge region. It is possible that such repeated dam-breach outburst floods played a crucial role in enabling fluvial erosion to keep pace with rapid rock uplift during the Quaternary.

### Role of the outburst flood in long-term landscape evolution

Although the 2000 Yigong outburst flood lasted only a few hours (Supplementary Fig. 11), our dataset suggests that it had significant impacts on surface processes throughout its evolution, resulting in several long-term consequences for regional landscape evolution. Firstly, when the landslide-dam breached, the outburst flood entrained large boulders by plucking from bedrock and remobilization of pre-existing deposits (Fig. 6) and brought a substantial amount of landslide sediment to the valley where hillslopes were undercut. Secondly, concurrent landslides induced by flood steepened hillslopes within the gorge and exposed more fresh bedrock (Supplementary Figs. 4, 8), causing further collapse of fractured bedrock and promoting additional landslides up to hundreds of meters above the valley floor (Supplementary Fig. 4). Thirdly, armoring of the riverbed with outsized boulders initially limited fluvial incision^60,61^, and produced cover-boulders which laterally regulated non-steady state channel configuration, promoting post-flood lateral erosion to hillslope and valley wall in mountain (Figs. 3, 4). The flood was a rapid and effective coarse-particle sorting process retaining large blocks while transporting finer sediment downstream into Tsangpo Gorge^11^ and towards the Bay of Bengal (Fig. 1a). In rapidly uplifting mountain belts, fluvial, glacial and hillslope erosion processes are closely linked^62,63^. Therefore, the complex patterns associated with short-lived extreme floods should exert a strong influence on more regular processes, promoting fluctuations between erosion-limited and transport-limited regimes.

Our simulation results, based on the pre-flood topography, demonstrate that the local high shear stress is not only associated with observed depositional legacy of the outburst flood, but also linked to channel avulsion within the valley floor (Fig. 6 and Supplementary Fig. 6). The magnitude of shear stress can serve as a proxy for quantifying geomorphic capability of these outburst floods, which are responsible for significant channel rearrangements. For instance, under high shear stress, areas with boulder bars experience erosion followed by deposition of boulders and blocks (Fig. 6). The extensive channel avulsion and straightening significantly influence the spatial distribution of shear stress and control the intricate process of erosion and deposition during the outburst flood. The post-flood channel is constrained by the boulder bars, therefore, it is difficult to experience large-scale oscillations under background flow unless the next outburst flood redistributes sediment and channel rearrangement again. Fluctuations in unstable river channels will further impact the erosion and sedimentation distribution of the next outburst flood. Outburst floods in Eastern Himalaya may have had a profound impact on shaping the rugged landscapes across the region, as in other high-topography or high-relief regions worldwide^52,64^.

## Conclusion

We utilize a combination of 2D flood hydraulic simulation and relevant geomorphic changes to reconstruct the valley evolution resulting from the interaction among flood dynamics, channel morphology, valley change and hillslope processes during the June 2000 Yigong outburst flood in the Eastern Himalaya. The eroded/exhumed volumes indicate an average erosion magnitude of approximate 10 m within the Tsangpo Gorge. This signifies that this rare and extreme outburst flood event, characterized by high shear stress (5–10 kPa), mobilized sediment accumulated over thousands of years of eroding activity. The imbalance between the recovery timescales associated with geomorphic impacts and the magnitude and frequency of such outburst floods along the Yarlung River has resulted in an unsteady state within the Tsangpo Gorge region. Powerful erosion potentially contributes to substantial deposition loads during flooding. Hydraulic simulations suggest that boulder bars may exert control over channel rearrangement, potentially promoting lateral erosion into marginal valley walls or hillslopes following the flood event, thereby influencing complex spatial patterns of erosion and deposition during subsequent outburst floods. We infer that the outburst flood, as the perturbation event with the background of the long-term fluvial evolution, exerts a profound erosion and deposition legacy in mountainous terrain.

### Supplementary Information

Supplementary Information.

## Data Availability

All data needed to evaluate the conclusions in the paper are present in the paper and/or the Supplementary Information.
