# Supplementary material for: Quantitative assessment of the erosion and deposition effects of landslide-dam outburst flood, Eastern Himalaya
Source: Sci Rep. 2024 Mar 25;14:7038. doi: 10.1038/s41598-024-57894-2 (PMC10963718; doi:10.1038/s41598-024-57894-2)
Supplement: Supplementary file 1 — Supplementary Information. [file 41598_2024_57894_MOESM1_ESM.docx]

Supplementary Information for

**Quantitative assessment of the erosion and deposition effects of landslide-dam outburst flood, Eastern Himalaya**

Xiaolu Dong^1^, Xianyan Wang^1^*, Long Yang^1^, Zhijun Zhao^2^*, Ronald Van Balen^3^, Xiaodong Miao^4^, Tao Liu^5^, Jef Vandenberghe^3^, Baotian Pan^6^, Martin Gibling^7^, Huayu Lu^1^

*Corresponding author. Email: [xianyanwang@nju.edu.cn (WANG](mailto:xianyanwang@nju.edu.cn%20(WANG) X.)

[zhaozhijun@nnju.edu.cn](mailto:zhaozhijun@nnju.edu.cn) (ZHAO Z.)

**This PDF file includes:**

Supplementary Text

Supplementary Figures 1 to 11

Supplementary Tables 1 to 3

Supplementary Text

**Background:**

**Geomorphic Setting.** The Eastern Himalaya is one of the most rapidly uplifting and eroding regions on Earth^14,18^. Here, the Yarlung River flows east about 1200 km along the Yarlung Tsangpo Suture Zone. Then it makes a nearly 180° turn to the south and cuts through the Tsangpo Gorge between two of the higher peaks and traverses the Himalaya. Finally, it flows to the Bay of Bengal as the Brahmaputra River (Fig. 1a)^14,33^. The Yarlung River delivers about 4000 m^3^/s of sediment and contributes about 50-70 % of the entire sediment load to the Brahmaputra River^12,15^, making it the largest sediment-source river in the Eastern Himalaya. The topographic evolution of the Eastern Himalaya may be largely controlled by the base level provided by the Yarlung River^65^. Of particular interest is the anomalously steep and narrow Tsangpo Gorge. This gorge is ~200 m wide, ~150 km long, and drops ~2 km (elevation from ~2900 m down to ~900 m). According to Turzewski. et al. (2019)^11^, there are at least 26 flood-related boulder bars (>100-m-scale) based on field surveys and Google Earth imagery.

In the Eastern Himalaya, outburst floods are common during the Quaternary, as evidenced by abundant geomorphic and sedimentary records^20,22,23,26-28,64,66-69^. The records consist of lacustrine sediment, remnants of dams, and paleoflood deposits. For example, near the entrance of the Tsangpo Gorge at the Eastern Himalaya, at least two Holocene outburst floods erupted from the Gega lake: a ~80 km^3^ paleolake produced peak discharges of up to 1×10^6^ m^3^/s at 1200-1600 yr B.P., and the other, ~832 km^3^ paleolake produced peak discharges of up to 5×10^6^ m^3^/s at 9000-10000 yr B.P. (Fig. 1b)^22^. Further upstream of the main stem^28,69-71^ and to the east in its tributaries^72,73^, sedimentary evidence for barrier lake outburst floods is visible at many sites.

**Study region.** The Yigong River, a tributary of the Parlung River, flows into the Yarlung River within the Tsangpo Gorge. The Yigong catchment lies within tectonically active mountain belts that contain the NW-SE trending Jiali strike-slip fault and the NE-SW trending Dongjiu-Milin strike-slip fault (Fig. 1b)^14,33^. Precipitation is high because the warm and humid Indian summer monsoon invades the Tibetan Plateau interior along the Yarlung Tsangpo valley^19^. The abundant moisture and high elevation result in one of the most extensive glacier coverages in central Asia^74^. The combination of seismic activity, high precipitation, and glacial avalanches in the Yigong River is likely to induce environmental threats that include earthquakes^75^, landslides, debris flows^76^, landslide dams and glacial lake outburst floods^77,78^.

The primary bedrock in the Yigong catchment consist of Permian limestone with conglomerate and slate, Neoproterozoic volcanic rock, phyllite, marble, schist, and gneiss. The Tsangpo Gorge is situated in Cretaceous ophiolites and migmatites and metamorphosed Permian phyllite, schist, limestone, and quartzite. The bedrock has well-developed fractures and is locally unstable. There are extensive hillslope areas with sediments on both sides of the valleys (Supplementary Fig. 8). Widespread Quaternary deposits, including debris fans, moraines, and flood deposits, are present.

**The 2000 Yigong landslide dam outburst flood.** The 2000 Yigong Lake outburst flood took place at the same location (30°10′44″N, 94°56′35″E) as the outburst flood in 1900^32^. At 20:00 on April 9, 2000, a huge landslide (Fig. 1c), triggered by excessive meltwater and rainfall^32,64^, occurred in Zhamu Creek, Yigong Town, Bomi County, blocking the main channel of the Yigong River, to form a dammed lake. At 19:00 on June 10, the lake overtopped and breached the blockage. The volume of the damming rockslide was about 3×10^8^ m^3^ (average height 60 m, length 2500 m, and width 2500 m)^32^. The impounded lake, known as Yigong Lake, had an average depth of 55 m, rising over two months from an initial ~2210 m.a.s.l. to ~2265 m.a.s.l., with a volume of 2×10^9^ m^3^. The peak discharge that reached Tongmai Bridge, 17 km downstream of the breach, was about 1.24 × 10^5^ m^3^/s^32^. Further, the flood traveled into the rugged Tsangpo Gorge on the east flank of the Namche Barwa massif and coursed down about 500 km to the Himalayan range front^31^.

The natural archives of the 2000 Yigong landslide and subsequent outburst flood are of global significance for geomorphic and geological hazard due to: (1) the landslide is one of the largest to have occurred worldwide since 1900; (2) it formed one of the largest landslide-dammed lakes in history and (3) the flood was the second largest landslide-dam outburst flood in recorded history, second only to the Great Indus River flood in 1841^31^, similar to the reported largest peak discharge from a constructed dam failure at Banqiao Dam in China on 7 August 1975^52^.

**Field and UAV survey:**

Field work includes the following three parts. First, the identification and measurement of boulders by field investigations to validate the accuracy of the UAV results. The boulder bars exhibited an imbricated structure with a certain degree of rounding, indicative of their exposure to flood flow rather than the gravitational forces associated with landslides. Second, on-site inspections were conducted to correlate the observed features of the landslide area with those depicted in the remote sensing images. Third, field observations further revealed evidence of deposition, notably in the formation of boulder bars, and erosion, including bedrock erosion, attributed to the Yigong outburst flood. These findings not only validate the impacts of the outburst flood but also contribute significantly to understanding the evolutionary processes associated with such events.

We utilized UAV data for two primary objectives. Firstly, as a high-resolution image source, it enabled us to enumerate the particle sizes within the boulder bars. Secondly, we employed the UAV-derived Digital Elevation Model (DEM) for a comparative analysis with widely used terrain data released pre and post-flood event (SRTM and ALOS). This comparison aimed to investigate potential discrepancies between these two datasets in capturing temporal changes in terrain, particularly crucial in high mountain canyon areas where DEM accuracy may be compromised.

**Reliability and verification of DOD results:**

In addition to the officially published DEMs (SRTM, ALOS), the photogrammetric Structure from the Motion technique was used to generate a 2019 DEM from high-resolution UAV photography. Comparison of the elevation data among SRTM, ALOS and UAV demonstrated that the SRTM DEM denotes accurately the topography before the flood, and the ALOS DEM denotes the topography after the flood. Although the terrain data we used is DSM, includes vegetation, the elevation of vegetation is within the error range of the datasets, which shows no significant impacts on our calculaitons on the topography changes pre- and post- the outburst flood. In addition, the differences between UAV and ALOS DEMs for valley cross-sections are small (Fig. 4). Thus, the resulting DOD calculations are reliable and can be used to investigate the erosive and depositional effects by the flood.

The active channel position at different times was determined using remote sensing images and DEM data. The channel has migrated since the flood in the section from the landslide dam to Tongmai Bridge (Fig. 4). The channel position extracted from the SRTM corresponds to the position based on the remote sensing images in 1999, whereas the channel position extracted from the ALOS data corresponds to the position based on the remote sensing images from 2001 to 2021 (Supplementary Fig. 1).

The valley upstream of Yigong Lake was not affected by the flood, which is confirmed by its DOD values. The frequency distribution histogram of DOD values within the upstream valley floor (Supplementary Fig. 3a) shows narrow peaks and small absolute values (within the range of ±3 m), basically within the error range. In contrast, the valley downstream of Yigong Lake was strongly affected by the flood and its DOD values vary considerably (Supplementary Fig. 3c): the elevation differences range from -60 to 30 m. Moreover, the number of grid cells with negative DOD values account for ~55% of the total, indicating that erosion dominated the flood.

The flood-induced concurrent landslides occurred in the downstream part of the Yigong River, where the DOD values are mostly negative, indicating erosion (Supplementary Fig. 3e). However, the DOD values of hillsides that were not affected by the flood in the lower reaches of Yigong River show a normal distribution, in the range of ±10 m (Supplementary Fig. 3d), similar to the DOD value distribution of the hillsides upstream of Yigong Lake (Supplementary Fig. 3b). The dispersion degree of the DOD values in non-affected regions (Supplementary Fig. 3a, b, d) is far less than that of the flood-affected regions (Supplementary Fig. 3c, e, f). The larger range of DOD values in the hillsides (Supplementary Fig. 3b, d) compared to the valley floor (Supplementary Fig. 3a) is mainly due to the lower vertical accuracy of topographic data in this terrain. We conclude that the computed DOD values reflect well the occurrence of concurrent landslides and topographic changes caused by the flood.

**Uncertainty of the DODs:**

Under the influence of terrain, the error distribution of the digital elevation data is not uniform. There method to calculate the error range of DOD, ***DOD data direct analysis,*** which is based on the DOD values of the non-affected geomorphic units. In addition, this error range is compared to the results of ***Error propagation analysis***, which is calculated based on the individual errors of DEMs^79-81^.

***DOD data direct analysis.*** We assume that for the terrain not affected by the flood (Supplementary Fig. 3a, b, d), the DOD value is close to zero within the acquisition time between the two DEM datasets (SRTM and ALOS). The error was calculated as follows:

$$\begin{aligned} RMSE=\sqrt{\frac{\sum_{i=1}^{n} \left( \delta z_{DOD} \right)^{2}}{n}}=\sqrt{\frac{\sum_{i=1}^{n} \left( z_{DOD}-z_{true} \right)^{2}}{n}}\#\left( S1 \right) \end{aligned}$$

where $\delta z_{DOD}$ is the error of the DOD for each cell; $n$ is the number of grid cells; $z_{DOD}$ is the value of DOD; $z_{true}$ is a constant, 0, the assumed true value of DOD for the non-affected flood region. The error in the valley floor is based on Supplementary Fig. 3a, resulting in the average root-mean-square-error (RMSE) of 5.39 m. Similarly, in the hillslope region (Supplementary Fig. 3b, d), the average root error (RMSE) is 10.05 m. The vertical accuracy of the valley floor elevation is higher than that of the hillside.

***Error propagation analysis.*** The propagated error was estimated from the following formula^79^:

$$\begin{aligned} \delta u_{DOD}=\sqrt{{\left( \delta z_{SRTM} \right)^{2}+\left( \delta z_{ALOS} \right)}^{2}} \#\left( S2 \right) \end{aligned}$$

where $\delta u_{DOD}$ is the propagated error in the DOD, and $\delta z_{SRTM}$ and $\delta z_{ALOS}$ are the errors in SRTM DEM and ALOS DEM. The RMSE of vertical accuracy of SRTM and ALOS in China is 5.86 m and 4.81 m^37^, respectively. For simplification, it is assumed that errors in each cell are random and independent^80^, and there is no spatially dependent error which might be untrue in this rugged topography. The estimated propagated error is 7.58 m (RMSE), which is within the range (5.39 – 10.05) of the results from the results of DOD data direct analysis.

Taking the simplified, *Error propagation analysis* as a reference, we suggest that the uncertainty of the DODs calculated by the *DOD data direct analysis* is reasonable.

**Dam break hydrograph:**

The dam break model was set up to get the outflow hydrograph of the breach based on the following information. The minimum height of the landslide-dam on Yigong Lake was about 55 m^31^. The landslide dam formed an extensive dammed lake^31^ with a maximum lake level of ~2265 m asl and an impounded volume of 2.015 Gm^3^. The elevation of the Yigong Lake at the upstream margin of the landslide-dam before the outburst flood is given as 2212 m asl by the SRTM data.

Before the simulation begins, we emplaced the dam between the upstream reservoir (Yigong Lake) and downstream 2D flow area on the relatively flat terrain, which makes the model stable. The initial conditions of the dam were set to infill the pre-flood valley with an elevation of 2265 m asl. Then we input the breach time, failure mode, and starting water surface to induce the dam break (Supplementary Table 3). (1) Failure mode is overtopping^31,64^ given that the rainfall supplement and the gradual increase of the water surface of the dammed Yigong Lake^32^. Overtopping refers to the reservoir water overflowing the dam crest, scouring and eroding the dam surface, and gradually developing a small rectangular (or other shape) breach. Then the rectangular breach expands downward and to the sides at a linear and uniform rate until the flow scouring is balanced with the dam material resistance or the reservoir water is completely drained. (2) The breach time is set to 0.1 hr assuming instantaneous collapse, which is the same as Turzewski et al. (2019)^11^. (3) Starting water surface is set to 2265 m asl, consistent with maximum lake level and the failure mode of overtopping. (4) Breach shape is set to an inverted trapezoid, and the height of the breach is 53 m considering a worst-case scenario with the landslide-dam breaches to its base of 2212 m asl. The final breach bottom width is 200 m, about half of the initial dam bottom width considering the post-flood topography of the breach. The boulders and blocks of the landslid-dam have not been transported by the lake water, and are located at the foot of the hillslope after the collapse. (5) The HEC-RAS dam break module uses the wide-crested weir outflow formula to simulate the outflow of the breach, and the formula^38^ is as follows:

$$\begin{aligned} Q=\delta_{s}\varepsilon mB\sqrt{2g}\left( Z_{i}-Z_{i+1} \right)^{\frac{3}{2}}\#\left( S3 \right) \end{aligned}$$

Where $Q$ is discharge; $\delta_{s}$is the submergence coefficient; $\varepsilon$ is the side shrinkage coefficient; $m$ is the discharge coefficient; $B$ is the width of broad-crested weir; $g$ is the acceleration of gravity; $Z_{i}$ and $Z_{i+1}$ are the water levels in front of and behind the weir, respectively.

**Evaluation of simulation results**

According to the simulation results (Supplementary Figs. 11), the flood propagation was as follows. The outburst occurred at 19:00 hr on June 10th, 2000, and 10 minutes later, the flow at the breach site reached ~ 14×10^4^ m^3^/s. After 20 minutes, the flood wave reached Tongmai Bridge. At 19:40 hr on June 10, the peak flow at Tongmai Bridge was ~ 12×10^4^ m^3^/s (Supplementary Fig. 11), and the maximum flow depth was ~66-68 m. Ten hours after the outburst, Yigong Lake was almost completely drained, and the discharge has been stable since then (Supplementary Fig. 11). The calculated peak discharges at the breach and Tongmai Bridge are slightly less than those calculated by Turzewski et al. (2019)^11^ and Morey et al. (2022)^25^using the GeoClaw model, which were 17.3×10^4^ m^3^/s and 13.9×10^4^ m^3^/s, respectively. In addition, Delaney and Evans (2015)^31^ estimated a peak discharge of 11×10^4^ m^3^/s at Tongmai Bridge from a synthetic hydrograph scaled to their estimated peak breach discharge of 61,461 m^3^/s, using the FLO-2D model. Zhuang et al. (2020)^82^ show that the maximum flow of the outburst flood at the Tongmai bridge was approximately 1.3×10^5^ m^3^/s exploiting the landslide dam modeling DAN_3_D and hydraulic model FLOW-3D. Moreover, Hu et al. (2021)^43^ reconstructed the water surface and energy surface during the Yigong outburst flood by the HEC-RAS 1D step-back water method given the flood discharge of 1.26×10^5^ m^3^/s. Furthermore, Zhang et al. (2022)^42^ input the calibrated hydrograph at the breach with longer time to reach the peak discharge (1.26×10^5^ m^3^/s) in the HEC-RAS as the initial condition using the 12.5 m ALOS DEM. The terrain data selected for the simulations, methods for obtaining the breach hydrograph and initial upstream boundary condition are different in these works. Our calculated results, first employing the instantaneous collapse of the dam and then implanting into 2D flood dynamics by HEC-RAS, are among the range of the results from previous work^11,25,31,42,43,82^. In addition, the peak discharge gradually decreases downstream (Supplementary Fig. 5), conforming to the basic law of outburst floods, supporting the validity of our hydraulic simulation.

The magnitudes of discharge, flow velocity, and other flood parameters were large compared to base flow. The calculated peak discharge of the flood was ~ 12×10^4^ m^3^/s, ~ 300 times the mean annual discharge, and more than 50 times the annual peak flood during the monsoon period^11,43^. The monsoon baseflow in this area can generate water depths of ~ 5-10 m^11,43^, whereas the Yigong flood could have been 50 m deep, and possibly 100 m deep, through the Tsangpo Gorge region (Supplementary Fig. 11). Here the outburst flood attained a velocity of around 20 m/s (Supplementary Fig. 11), which is twice the measured peak velocities (i.e., 10 m/s) in gorges of the Indus River^52^.

**Thresholds of shear stress**

Our simulation produces a spatial assessment of the bed shear stress. Here, we estimated thresholds of shear stress within Tsangpo Gorge for incipient motion, plucking a protruding block via sliding, and suspension using the calculation formula in previous work^4,9,11,21,83^. Results show the relationship between the given particle size and the thresholds of shear stress for different erosion processes (Fig. 6).

***The threshold shear stress for incipient motion*** was calculated^9^ using:

$$\begin{aligned} \tau_{c}^{*}=0.15S^{0.25}\#\left( S4 \right) \end{aligned}$$

$$\begin{aligned} \tau_{b}=D\times\tau_{c}^{*}\times g\times(\rho_{s}-\rho)\#\left( S5 \right) \end{aligned}$$

where $\tau_{c}^{*}$ is the dimensionless critical shear stress for incipient motion; $S$ is the river gradient (set to 0.01); $\tau_{b}$ is the threshold shear stress for movable blocks; $D$ is the median block size; $g$ is gravitational acceleration; $\rho_{s}$ is the density of granite (2700 kg/m^3^); and $\rho$ is the density of water (1000 kg/m^3^).

***The threshold shear stress for suspension*** was calculated^11^ using:

$$\begin{aligned} R=\frac{(\rho_{s}-\rho)}{\rho}\#\left( S6 \right) \end{aligned}$$

$$\begin{aligned} w_{s}=\frac{RgD^{2}}{{c_{1}v+(0.75c_{2}RgD^{3})}^{0.5}}\#\left( S7 \right) \end{aligned}$$

$$\begin{aligned} \tau_{b}={\rho(0.8w_{s})}^{2}\#\left( S8 \right) \end{aligned}$$

where $w_{s}$ is the setting velocity; $c_{1}$ is the grain shape, an empirical constant of 20; $c_{2}$ is roughness, an empirical constant of 1.1; $v$ is the kinematic viscosity, an empirical constant of 10^-6^ m^2^/s; $\tau_{b}$ is the threshold shear stress for suspended deposits.

***The threshold shear stress for block plucking*** was calculated^83^ using:

$$\begin{aligned} \tau_{pc}^{*}=\frac{\cos\theta\left( \tan\varphi-\tan\theta\right)+2\tau_{w}^{*}}{\left[ 1+0.5C_{d}\left( \frac{u}{u^{*}} \right)^{2}\frac{P}{L} \right]\left[ 1+F_{L}^{*}\tan\varphi\right]}\#\left( S9 \right) \end{aligned}$$

$$\begin{aligned} \tau_{pc}=D\times\tau_{pc}^{*}\times g\times(\rho_{s}-\rho)\#\left( S10 \right) \end{aligned}$$

where $\tau_{pc}^{*}$ is the dimensionless critical shear stress for plucking; $\theta$ is the bed angle (set to 1°); $\varphi$ is the bed friction angle (set to 35°); $\tau_{w}^{*}$ is dimensionless block sidewall stress (set to 0); $C_{d}$ is the local drag coefficient (set to 1); $P$ is the block protrusion height (0.1 or 0.2); $L$ is the block length; $F_{L}^{*}$ is the dimensionless hydraulic lift force (set to 0.85); and $\frac{u}{u^{*}}$ is 8.3, the same as in Turzewski et al. (2019)^11^; $\tau_{b}$is the threshold shear stress for plucking. Here, we notice that the bed angle $\theta$ has little influence, with the critical shear stress changing slightly (3%) when the bed angle changed from 0.5° to 1.5°. We measured several bed angle values from DEM and finally set the median at 1°.

**Erosional impact of the outburst flood compared with long-term denudation**

The study area lies in the Namche Barwa-Gyala Peri (NBGP) massif, where a close coupling between tectonics and climate governs surface processes, leading to erosion through river incision and landslides. Assessment of the 2000 Yigong outburst flood is based on the volume and area of alluvial sediment and bedrock eroded across the valley floor during the 2000 outburst flood, combined with the volume and area of concurrent landslides on the adjoining hillslopes. For the valley floor, we calculated the difference between digital elevation maps before and after the flood (DOD) along the ~ 80 km reach downstream from the dam breach. The assessment takes into account erosion and deposition across the whole valley floor affected by the flood, yielding an equivalent **mean erosion depth**. For concurrent landslides above the valley floor, we used the landslide erosion volume divided by the landslide area, yielding an equivalent **mean erosion depth**. Based on their respective areas along the reach, the depths were integrated to calculate the **equivalent average erosion depth** for the 2000 outburst flood in the study area. The study reach lies directly below the breached landslide dam. Consequently, we infer that the bulk of the deposited material originated in the study reach, allowing a robust calculation of erosion and deposition in the reach. However, some deposits may have been swept into the reach from the lake bed and margin upstream.

Several methods have yielded denudation rates over different timescales for the NBGP massif, and they are generally in agreement with each other, despite some discrepancies. Thermochronological methods sampling bedrock or detrital sediment provide estimates averaged over ~ 10^6^ years and have yielded a denudation rate of ~ 5-10 mm/yr^12,14,16,17^. Cosmogenic radionuclide methods provide estimates averaged over ~ 10^3^ years and have yielded a denudation rate of 4-28 mm/yr^13^. Suspended sediment load of rivers within the catchment provides estimates for annual to decadal timescales and has yielded an average annual denudation rate for the 1971-1979 period of 10 mm/yr (5-17 mm/yr)^12,15^. The denudation rates obtained by the different methods have limitations. Modern suspended sediment loads may be affected by enhanced sediment flux due to anthropogenic effects, the stochastic nature of precipitation, and by the fact that only the suspended sediment load is used and not the total sediment load. Cosmogenic radionuclide data have a large error range due to active landslides along the Tsangpo Gorge^13^, and the time scale of the thermochronological methods is long. Nevertheless, the values accord broadly.

The present study considers erosion over the duration of the Quaternary. Therefore, in order to calculate how many years of regular meteorological processes might be needed to generate an equivalent amount of total erosion, we apply denudation rates based on thermochronological and cosmogenic data. The equivalent average erosion depth for the 2000 flood was divided by a range of 5 mm/yr to 10 mm/yr.

**
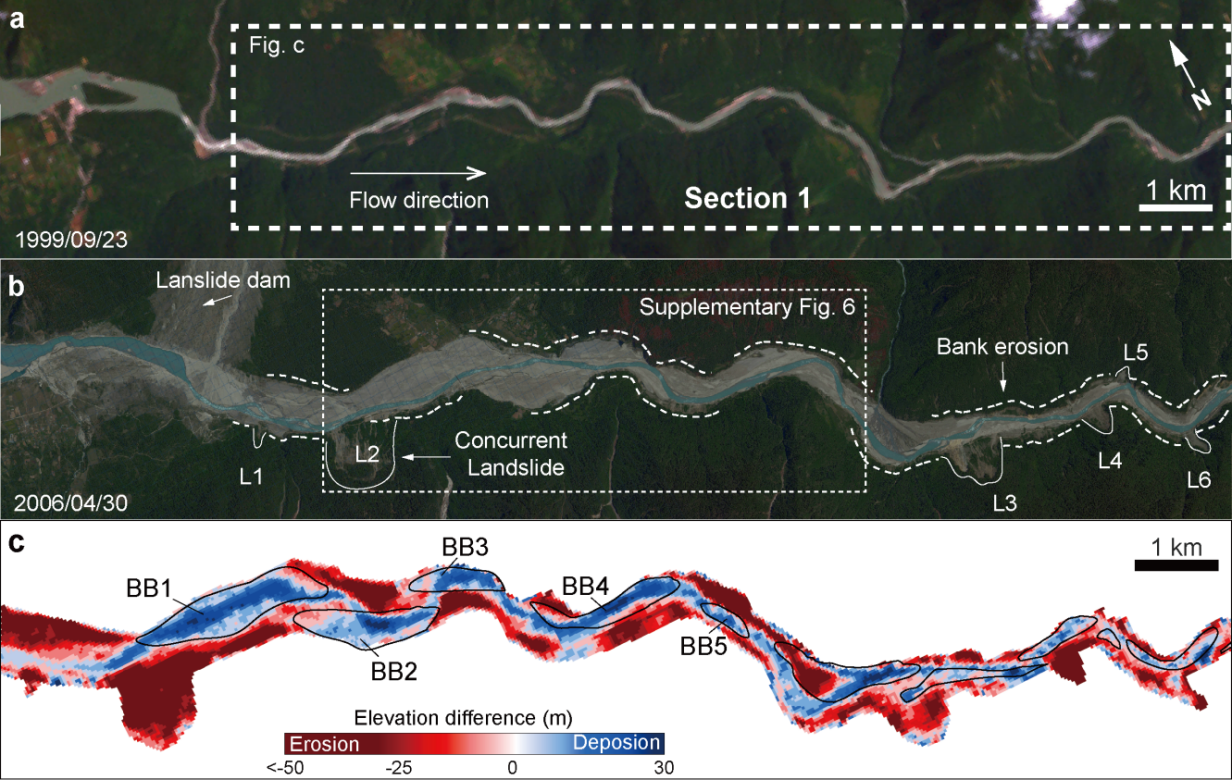
**

**Supplementary Fig. 1.** The landscape changes by comparing the images before and after the flood in Section 1 (see Fig. 1c). **a**, Landsat 7 ETM＋image (https://earthexplorer.usgs.gov/) obtained on September 23, 1999, showing the pre-flood topography. **b**, Google Earth image (https://earth.google.com/web) on April 30, 2006, showing bank erosion and concurrent landslides (L1-L6) due to lateral erosion. Along half of the channel migration reach, the post-flood channel is controlled by coarse boulder bars on one bank and contact with the steep valley walls on the other bank. **c**, Enlarged view of DOD map for Section 1. The maps were created using a licensed ArcGIS 10.2 software (https://support.esri.com/zh-cn/overview).


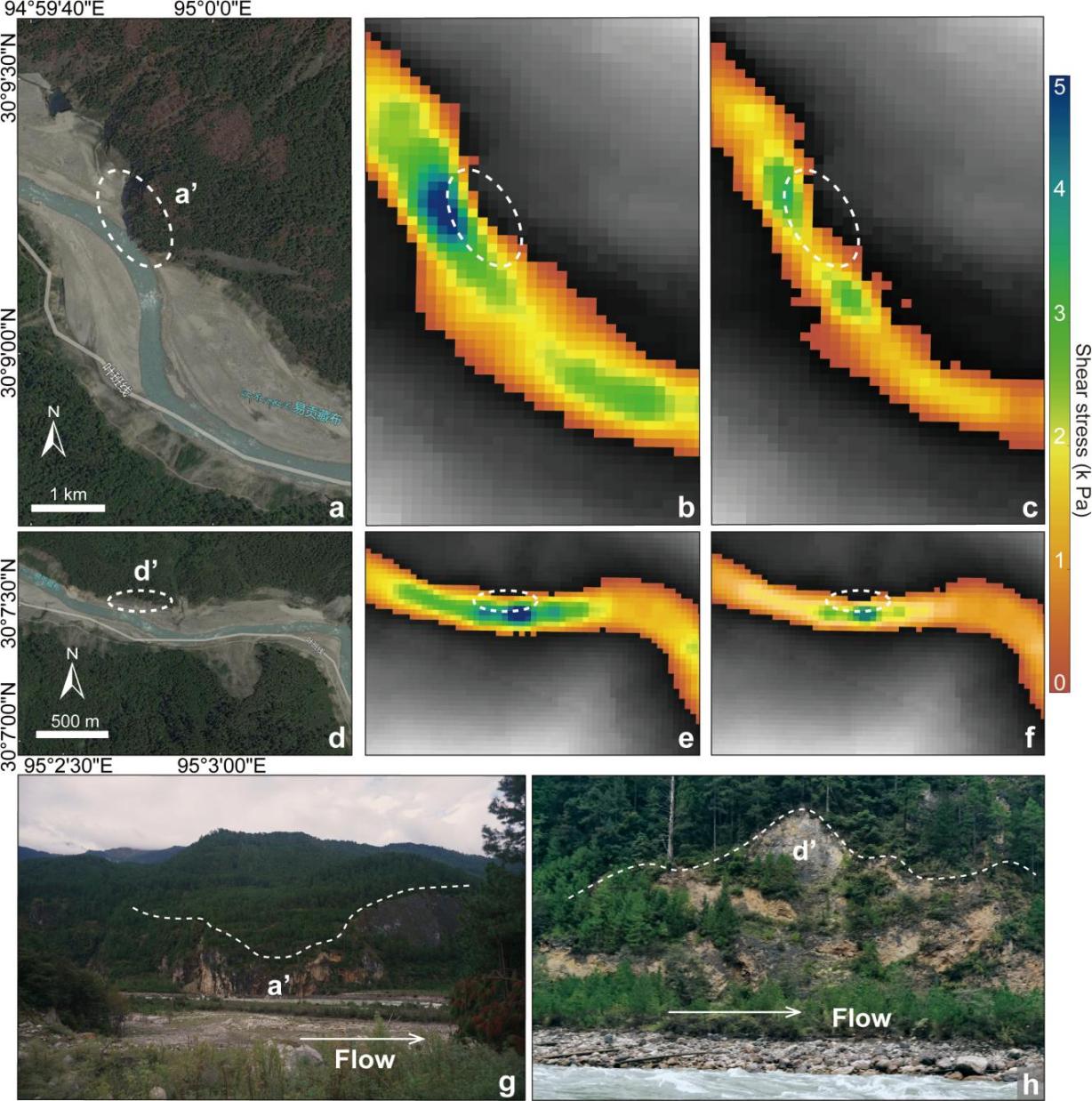


**Supplementary Fig. 2.** Lateral erosion by the flood and the distribution of shear stress. **a**, **d**, Google Earth images (30 April 2006, https://earth.google.com/web) showing valley constriction. **g**, **h**, Field photographs taken in October 2021 showing the valley side eroded to the bedrock, where a’ and d’ are located in Supplementary Fig. 2a, d. Simulated shear stress at t = 0.5 (**b**), 1 (**e**), and 3.5 (**c**, **f**) hours after the breach. High shear stresses (2-5 kPa) last for at least 3 hours during the flood, which could pluck (Fig. 6, see Methods) and abrade^11,26^ the valley wall. The maps were created using a licensed ArcGIS 10.2 software (https://support.esri.com/zh-cn/overview).


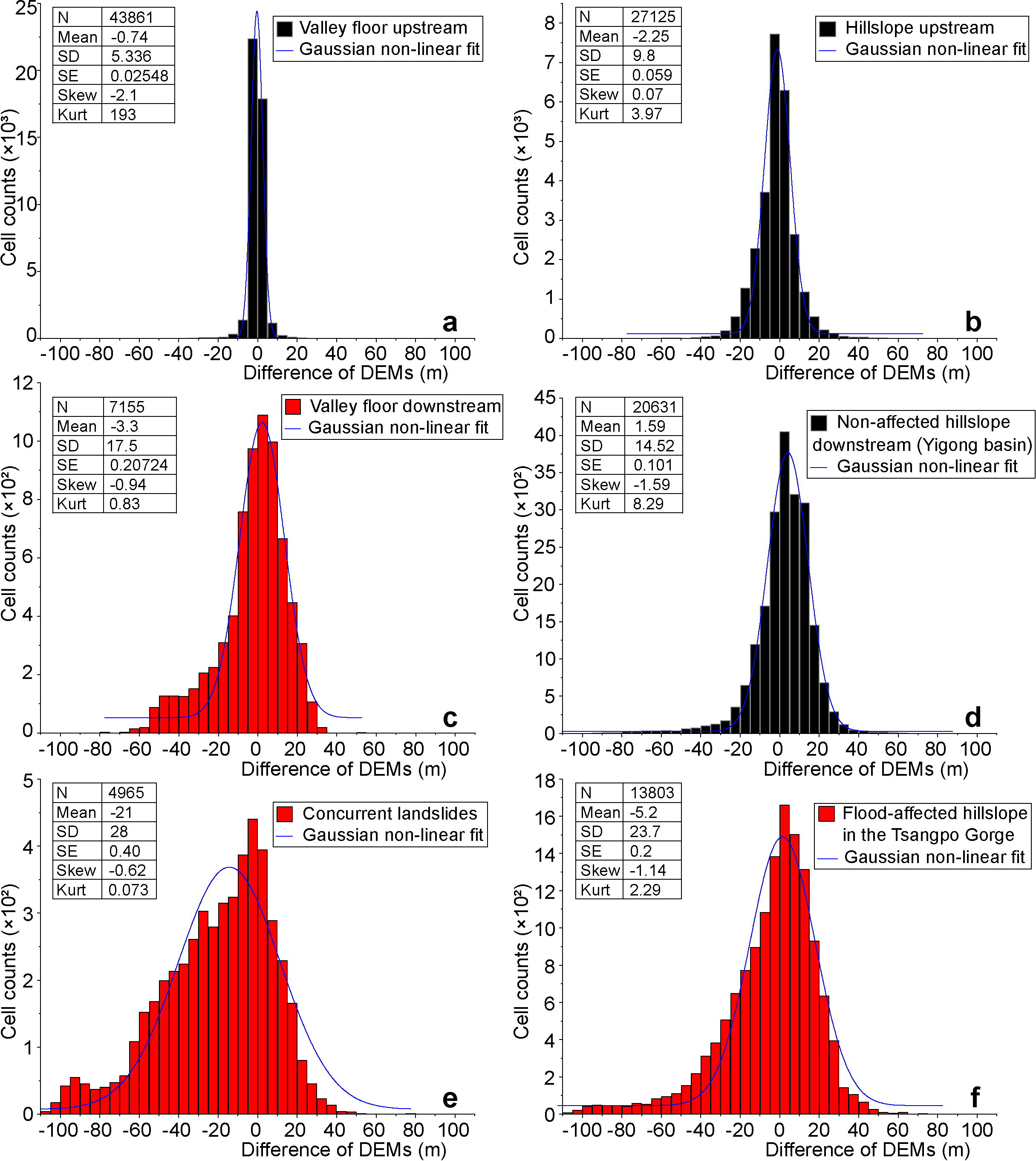


**Supplementary Fig. 3.** Validation of DOD results. DOD values of grid cells are counted to compare the distribution of elevation differences in areas of the upstream valley floor (**a**), upstream hillslope (**b**), downstream valley floor (**c**), downstream non-affected hillslope (**d**), concurrent landslides (**e**) and flood-affected hillslopes in the Tsangpo gorge (**f**). See locations in Supplementary Fig. 10. Red bars (a, b, d) designate flood-affected features and black bars (c, d, e) denominate features that are not affected by the flood.


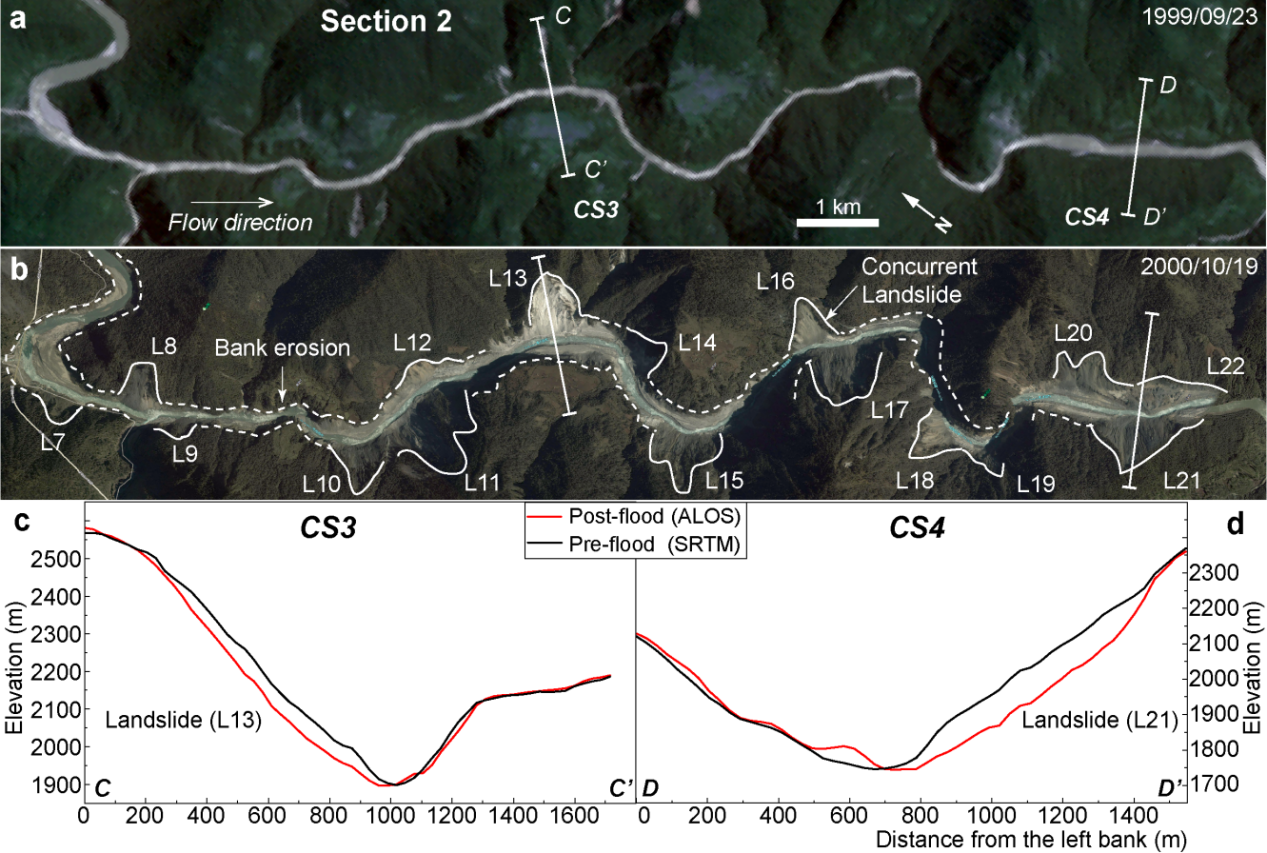


**Supplementary Fig. 4.** Geomorphic effects of the flood along the Tsangpo Gorge in Section 2 (see location at Fig. 1c). **a**, Landsat 7 ETM＋ image (https://earthexplorer.usgs.gov/) obtained on September 23, 1999, as the pre-flood topography. **b**, Google Earth image (https://earth.google.com/web) on October 19, 2000, showing bank erosion and resulting concurrent landslides (L7-L22) caused by the flood. **c**, **d**, Elevation profiles of the cross sections (CS3 and CS4, see locations in Supplementary Fig. 4a. The valley was significantly widened by concurrent landsliding (Supplementary Fig. 4b). In this way large amounts of mass have been transported downstream by the flood. The maps were created using a licensed ArcGIS 10.2 software (https://support.esri.com/zh-cn/overview).


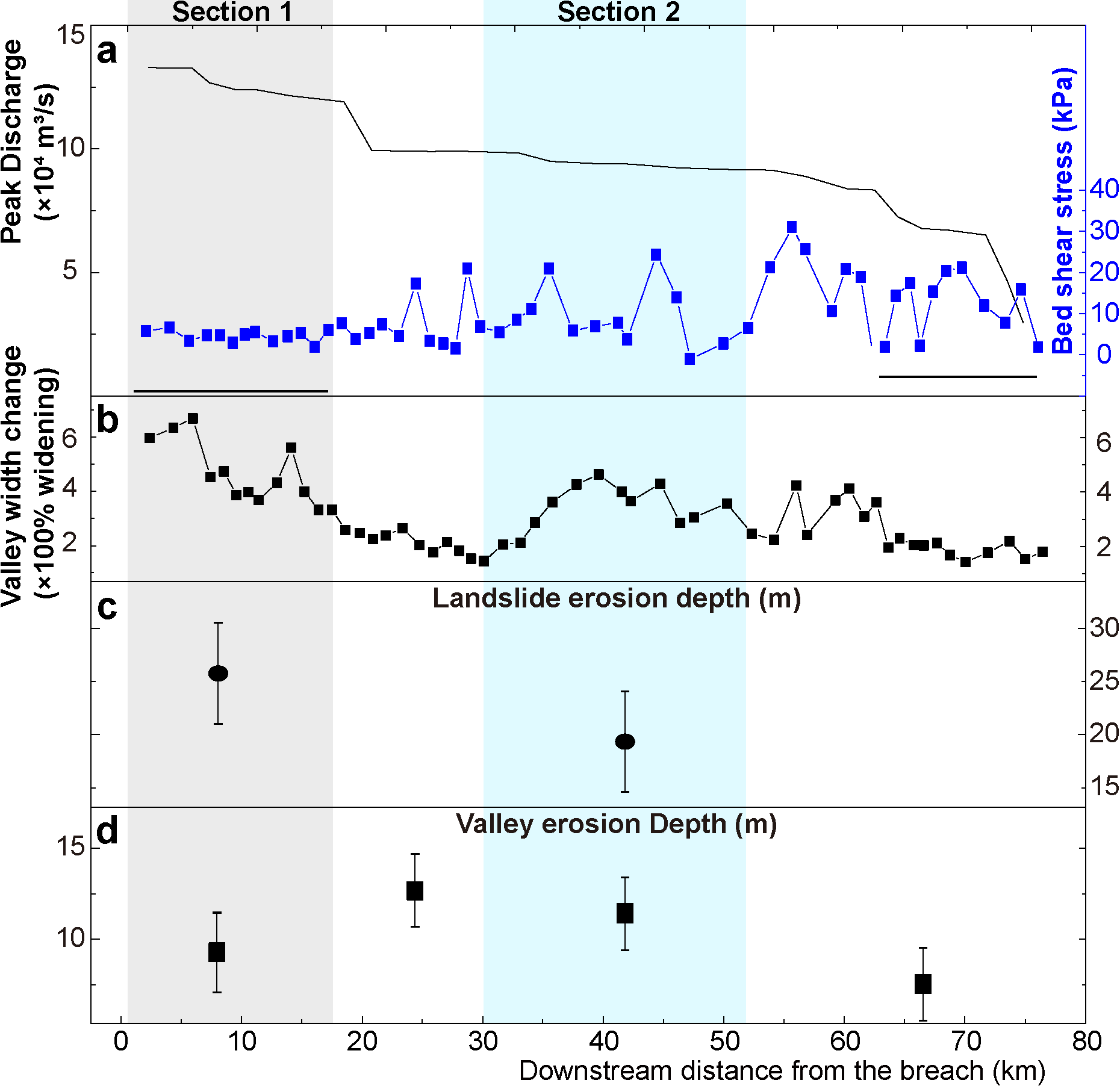


**Supplementary Fig. 5.** Erosion by the flood based on the HEC-RAS model and DOD calculations. **a**, Hydraulic factors (maximum shear stress, and maximum discharge). The black horizontal lines are the annual discharges in different areas. **b**, Changes of valley-floor width from the breach to ~80 km downstream. **c**, **d**, Calculated range of flood-induced landslides erosion depth (c) and valley erosion depth (d) in specific parts of the channel.


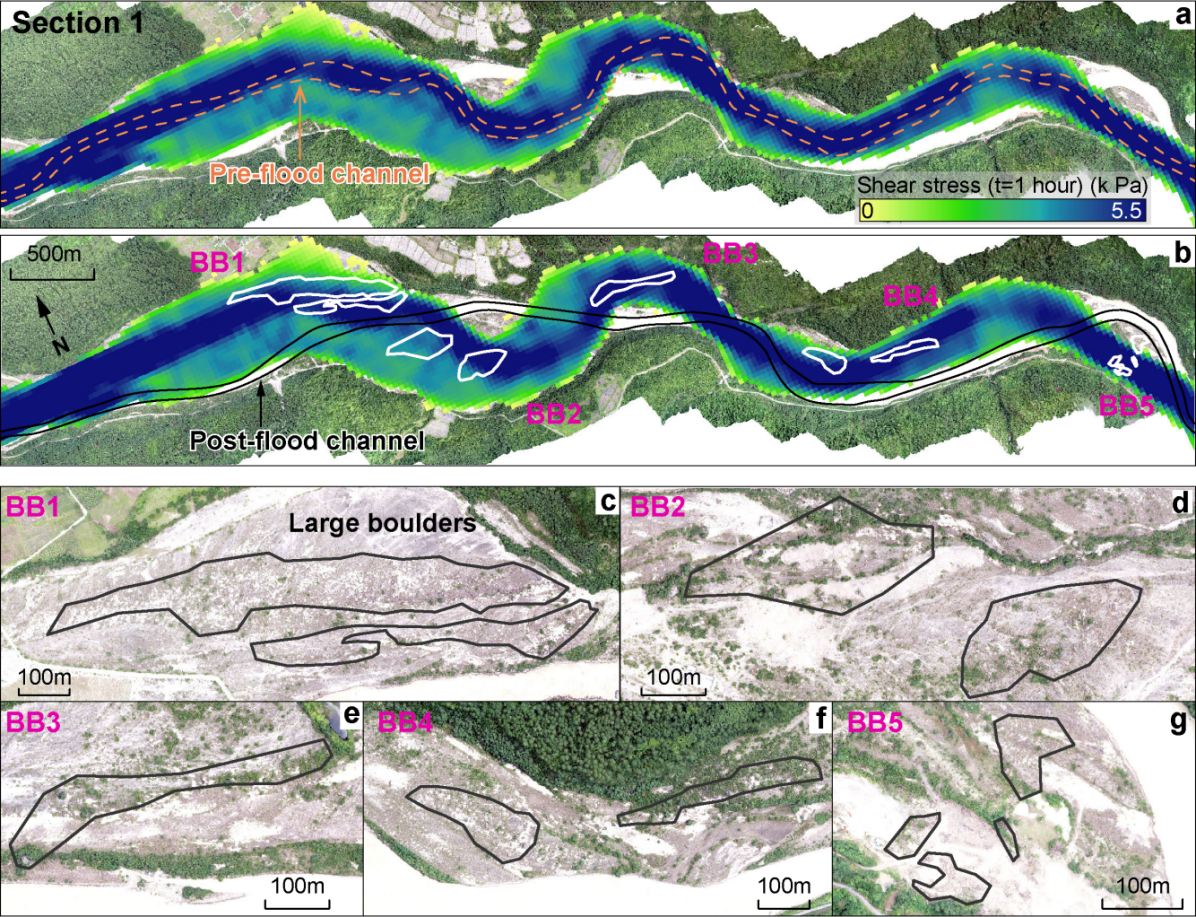


**Supplementary Fig. 6.** Landforms accumulated by the flood (shear stresses and boulder bars). **a**, Correspondence between the distribution of shear stress one hour after the breach and the pre-flood channel. The highest shear stress occurred at the location of the pre-flood channel. The extent is indicated in Supplementary Fig. 1b. **b**, The relationship between the flow shear stress distribution at 1 hour after the outburst and the location of current channel, boulder bars, and large boulders and blocks. **c**-**g**, Aerial photographs of boulder bars B1-BB5.


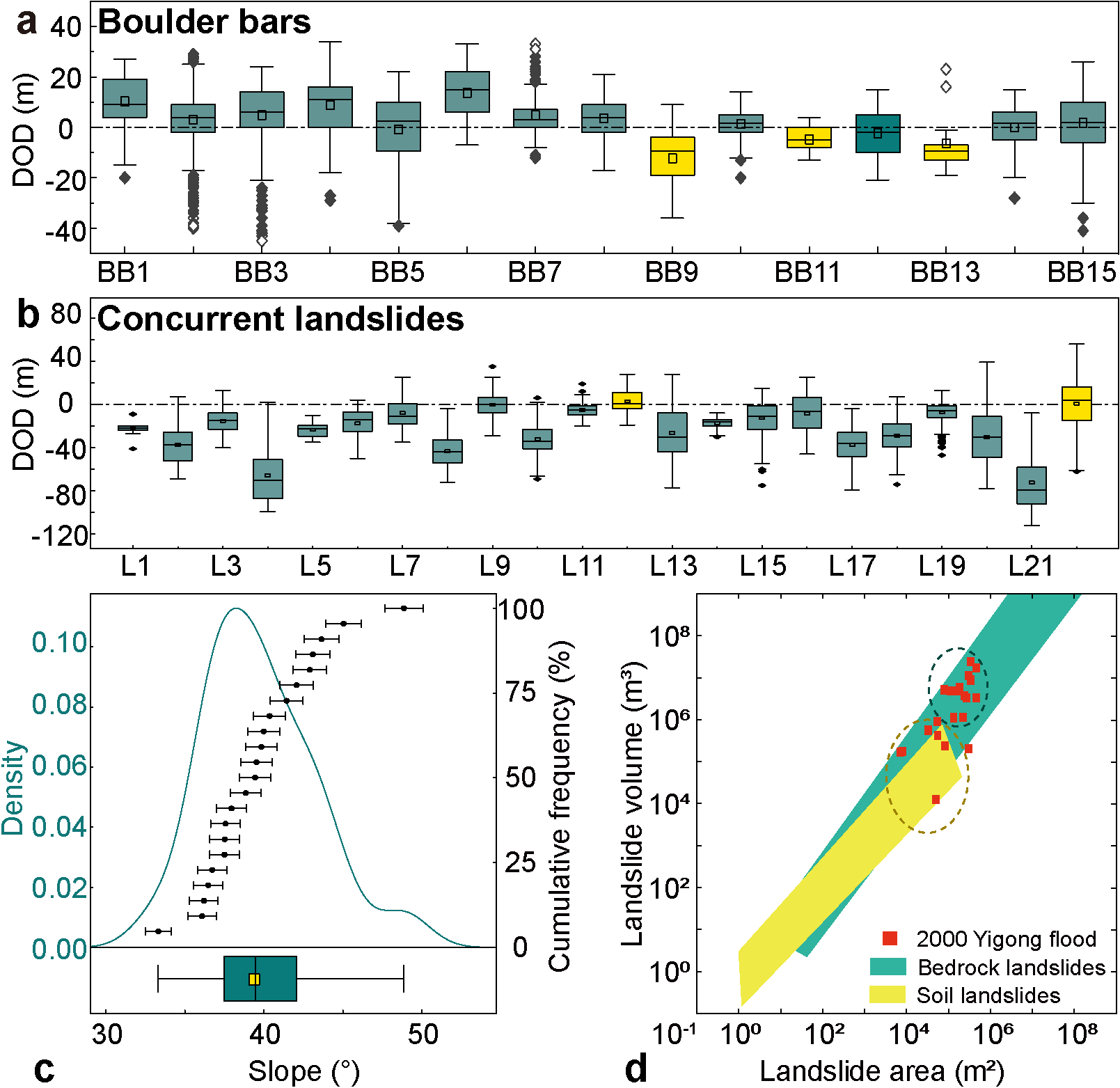


**Supplementary Fig. 7.** The DOD results of boulder bars and concurrent landslides. **a**, Distribution of DOD values for each boulder bar (BB1-BB15, see locations at Fig. 2). The grayish green bars indicate the boulder bars which were accumulated by the flood. The yellow bars indicate the boulder bars that were eroded by the flood. The box spans the interquartile range, the black dot denotes the average, the line denotes the median, 25th to 75th percentiles and whiskers denote Q3±IQR (outlier truncation point). **b**, Distribution of DOD values for each concurrent landslide (L1-L22, see locations at Fig. 2). The grayish-green bars indicate the location of landslides that were eroded. The yellow bars indicate the location of landslides that were accumulated. **c**, Kernel density estimate plot of the gradient of concurrent landslides. The box at the bottom spans the interquartile range, the yellow dot denotes the average, the line denotes the median, 25th to 75th percentiles, and whiskers denote Q3±IQR. The numbered boulder bars and concurrent landslides are located as shown in Fig. 2. **d**, Area-volume relationship of concurrent landslides. The blue and yellow polygons represent Himalayan landslides in solid bedrock and soil, respectively, based on data from^24,52^. The red solid dots represent flood-induced concurrent landslides calculated from remote sensing images and DOD data in this study.


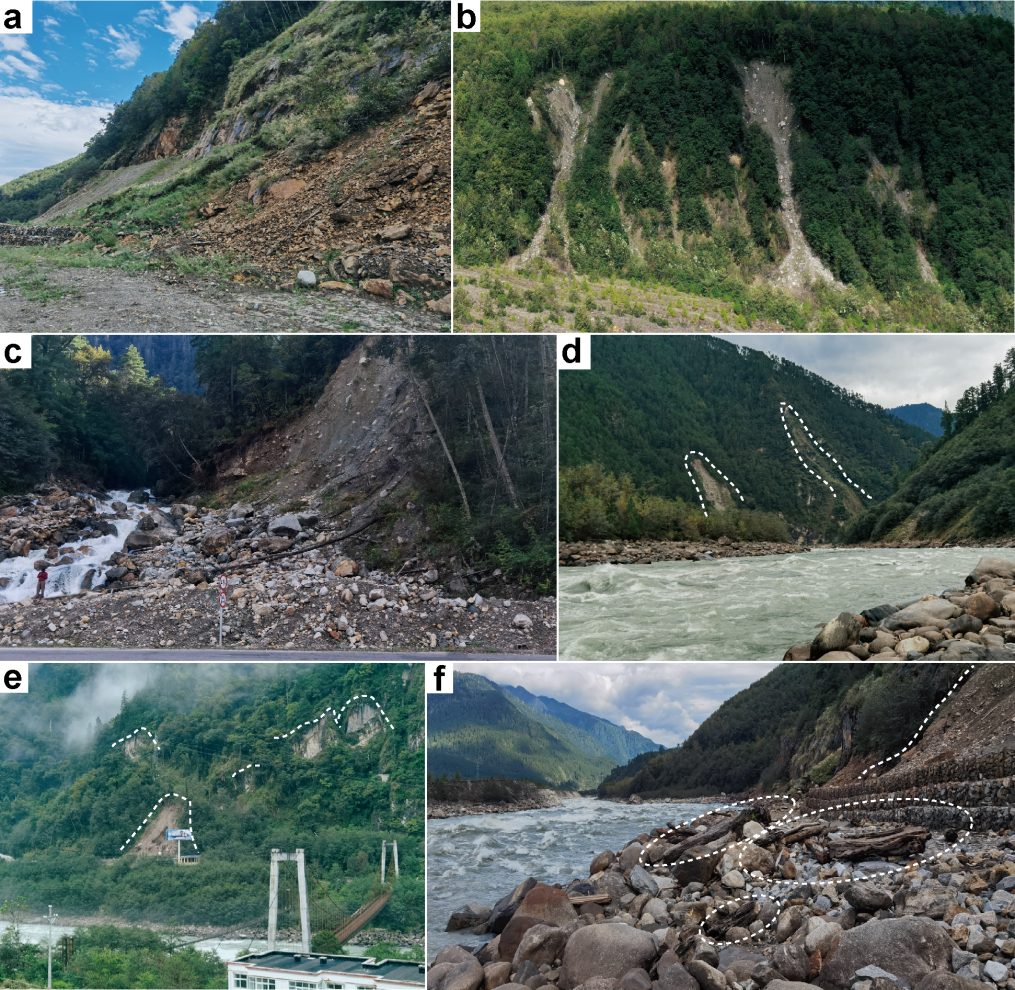


**Supplementary Fig. 8.** Bank erosion (a, c, f), concurrent landslides (b, d, e), and branches from trees destroyed and transported (f) by the flood.


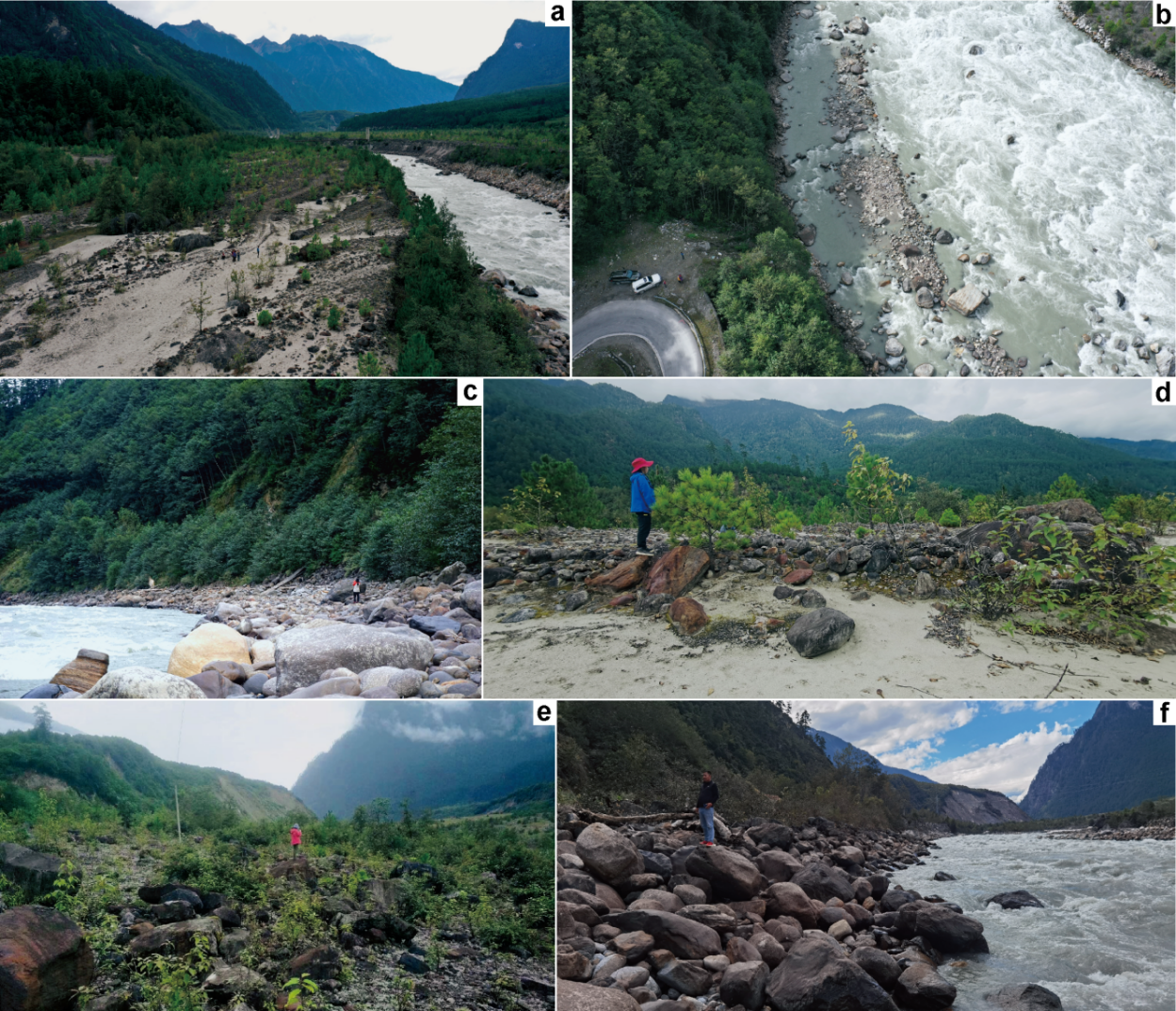


**Supplementary Fig. 9.** Boulder bars deposited by the 2000 Yigong outburst flood showing imbricated, sub-rounded, and poorly sorted boulders and blocks. Boulders and blocks (a, b) are located near the landslide dam.


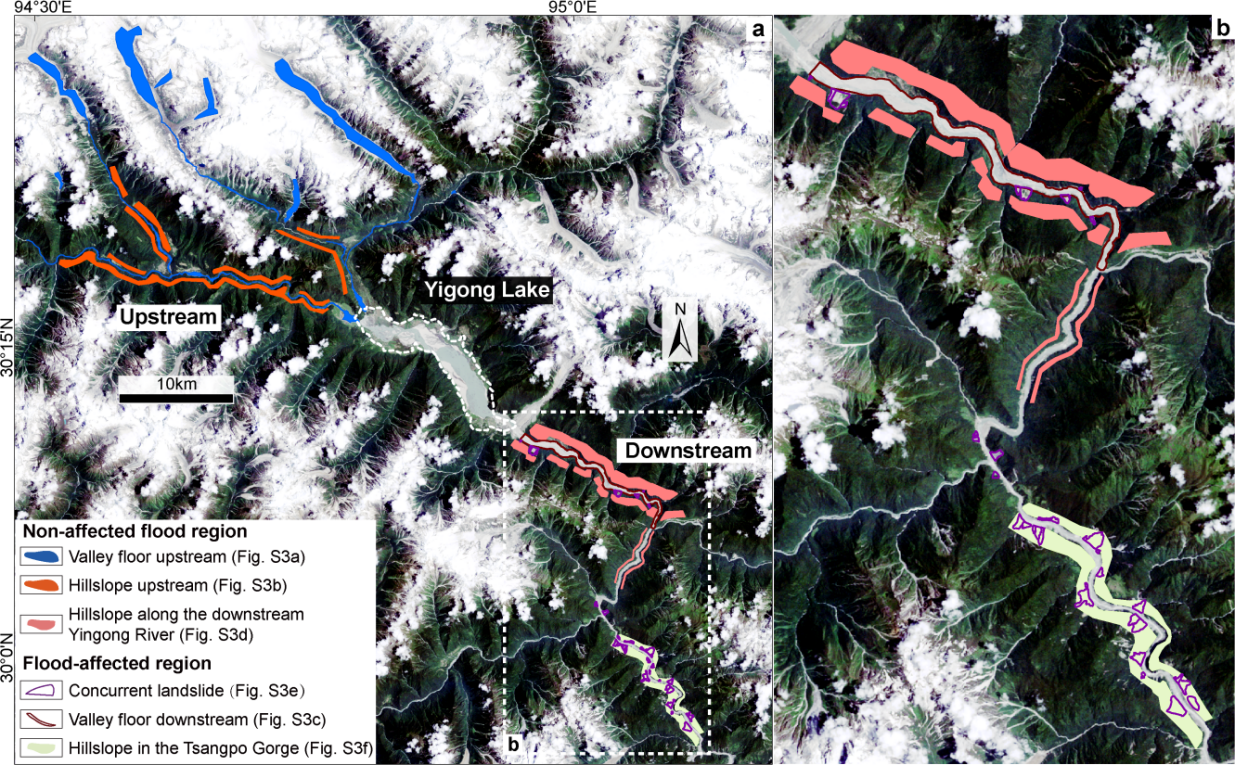


**Supplementary Fig. 10.** Range of DOD verification. Distribution of DOD values for the regions in Supplementary Fig. 3. The maps were created using a licensed ArcGIS 10.2 software (https://support.esri.com/zh-cn/overview).


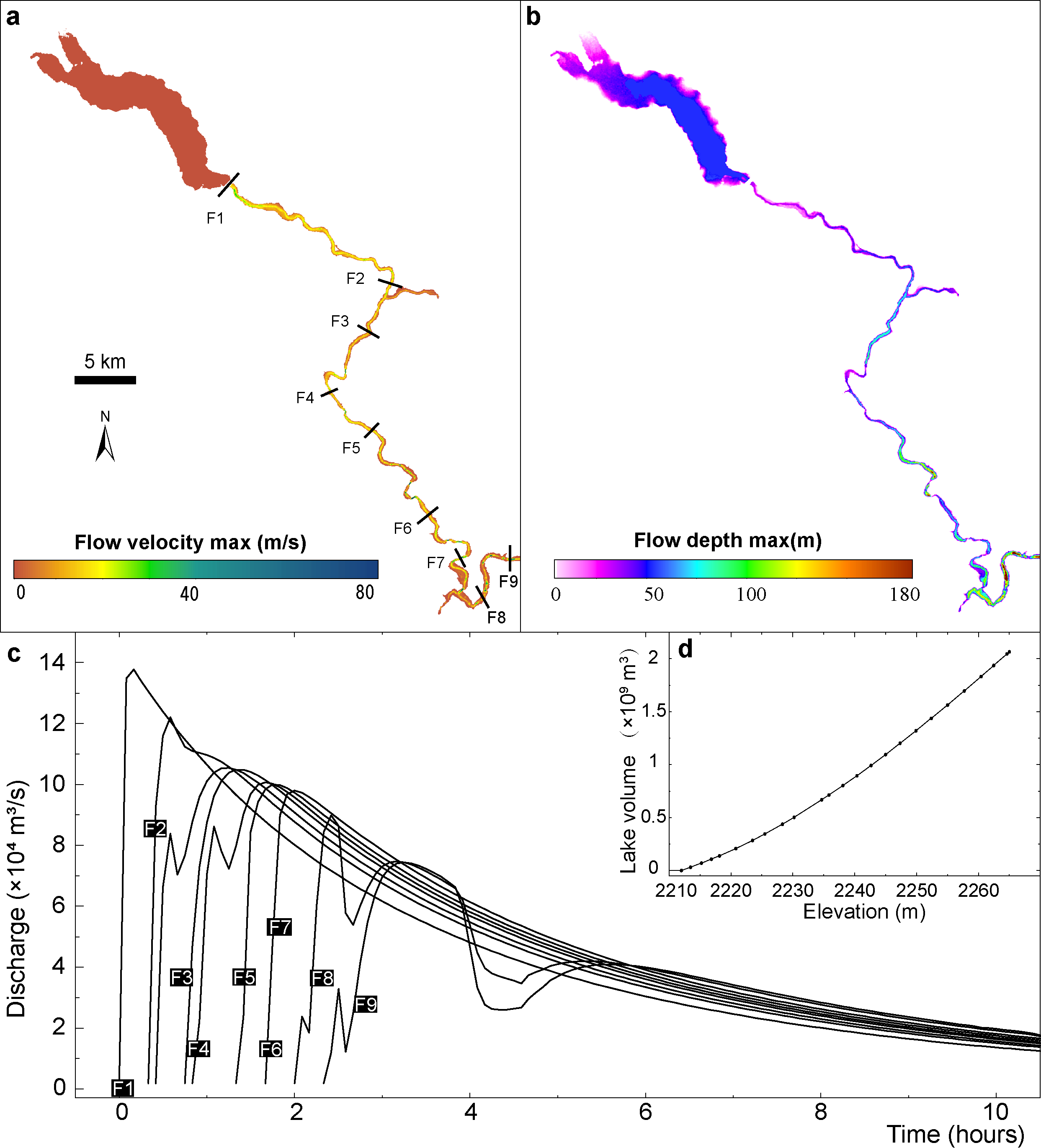


**Supplementary Fig. 11.** Results of the 2D HEC-RAS simulation. **a**, **b**, Maximum flow velocity and depth during the flood. **c**, Simulated discharge evolution at sites F1-F9 (see location in Supplementary Fig. 11a) during the first 10 hours of the flood, in which F1 and F2 show discharge recorded at the breach and Tongmai Bridge from simulations, respectively. **d**, Elevation-volume curve of Yigong Lake from HEC-RAS results, assuming a maximum lake level elevation of ~2265m a.s.l.

**Supplementary Table 1.** Detail information about the airports around the Yigong Lake

| Location | Airport | Elevation / m a.s.l. | | |
| --- | --- | --- | --- | --- |
|  |  | ALOS | SRTM | Official registered |
| 97°6'32.4"E, 30°33'14.4"N | Qamdo Bangda Airport | 4336 | 4341 | 4334 |
| 94°20'2.4"E, 29°18'10.8"N | Nyinchi Airport | 2953 | 2951 | 2949 |
| 90°54'43.2"E, 29°17'52.8"N | Gonggar Airport | 3572 | 3570 | 3570 |

**Supplementary Table 2.** Digital topographic data and satellite imagery used in this study

| Type | Source | Acquisition date | Time node |
| --- | --- | --- | --- |
| Image^1^ | Landsat 5 | 1993/5/25 | Pre-flood |
|  | Landsat 5 | 1996/4/15 | Pre-flood |
|  | Landsat 7 | 1999/9/23 | Pre-flood |
|  | Landsat 7 | 2000/5/4 | Pre-flood |
|  | Landsat 5 | 2000/5/12 | Pre-flood |
|  | Landsat 5 | 2000/7/15 | Post-flood |
|  | Landsat 7 | 2001/3/20 | Post-flood |
|  | Landsat 7 | 2002/10/17 | Post-flood |
|  | Landsat 5 | 2003/7/24 | Post-flood |
|  | Landsat 5 | 2004/5/7 | Post-flood |
|  | Landsat 7 | 2006/7/8 | Post-flood |
|  | Landsat 5 | 2007/4/30 | Post-flood |
|  | Landsat 5 | 2008/7/5 | Post-flood |
|  | Landsat 5 | 2010/3/21 | Post-flood |
|  | Landsat 7 | 2011/8/23 | Post-flood |
|  | Landsat 7 | 2012/5/5 | Post-flood |
|  | Landsat 8 | 2013/8/4 | Post-flood |
|  | Landsat 8 | 2014/3/16 | Post-flood |
|  | Landsat 8 | 2015/7/25 | Post-flood |
|  | Landsat 8 | 2016/7/27 | Post-flood |
|  | Landsat 8 | 2017/2/4 | Post-flood |
|  | Landsat 8 | 2019/6/2 | Post-flood |
| DEM^2^ | SRTM | 2000/2/2 | Pre-flood |
|  | ALOS | 2006- | Post-flood |

^1^ The images (30-m resolution) are used to generate landslide inventories and quantify valley width.

^2^ The DEMs (30-m resolution) are used to calculate terrain change (volume and depth of erosion, and accumulation by the flood).

**Supplementary Table 3.** Input parameters about the dam break model

| Scenario | Parameters | Information |
| --- | --- | --- |
| Dam | The centerline of the dam | 94°55′53.56″ E; 30°10′53.74″ N |
|  |  | 94°56′06.10″ E; 30°11′04.12″ N |
|  | Weir Top Elevation | 2265 m asl |
|  | Weir Top Width | 461.37 m |
|  | Weir crest shape | Broad crested |
|  | Weir coefficient (C_d_) | 1.66 |
| Breach | Final Bottom Elevation | 2212 m asl |
|  | Final Bottom Width | 200 m |
|  | Left side slope | 2 |
|  | Right side slope | 2 |
|  | Breach Weir Coef | 1.44 |
|  | Breach Formation Time | 0.1 hrs |
|  | Failure mode | Overtopping |
|  | Starting water surface (WS) | 2265 m asl |

**Supplementary References：**

1. Schmidt, J. L., Zeitler, P. K., Pazzaglia, F. J., Tremblay, M. M., Shuster, D. L. & Fox, M. Knickpoint evolution on the Yarlung river: Evidence for late Cenozoic uplift of the southeastern Tibetan plateau margin. Earth and Planetary Science Letters 430, 448-457 (2015).
2. Huang, S.-Y. et al. Late Pleistocene sedimentary history of multiple glacially dammed lake episodes along the Yarlung-Tsangpo river, southeast Tibet. Quaternary Research 82, 430-440 (2014).
3. Panda, S. et al. Chronology and sediment provenance of extreme floods of Siang River (Tsangpo‐Brahma putra River valley), northeast Himalaya. Earth Surface Processes and Landforms 45, 2495-2511 (2020).
4. Srivastava, P. et al. Paleofloods records in Himalaya. Geomorphology 284, 17-30 (2016).
5. Yang, A. et al. Two megafloods in the middle reach of Yarlung Tsangpo River since Last-glacial period: Evidence from giant bars. Global and Planetary Change, 208 (2022).
6. Hu, H. P., Feng, J. L. & Feng, C. Sedimentary records of a palaeo-lake in the middle Yarlung Tsangpo: Implications for terrace genesis and outburst flooding. Quaternary Science Reviews, 192, 135-148 (2018).
7. Kaiser, K., Lai, Z., Schneider, B. & Junge, F. W. Late Pleistocene genesis of the middle Yarlung Zhangbo Valley, southern Tibet (China), as deduced by sedimentological and luminescence data. Quaternary Geochronology 5, 200-204 (2010).
8. Wang, H., Cui, P., Liu, D., Liu, W. & Lei, Y. Evolution of a landslide-dammed lake on the southeastern Tibetan Plateau and its influence on river longitudinal profiles. Geomorphology, 343, 15-32 (2019).
9. Wang, H., Wang, P., Hu, G., Ge, Y. & Yuan, R. An Early Holocene river blockage event on the western boundary of the Namche Barwa Syntaxis, southeastern Tibetan Plateau. Geomorphology 395 (2021).
10. Hu, G., Yi, C.-L., Zhang, J.-F., Liu, J.-H. & Jiang, T. Luminescence dating of glacial deposits near the eastern Himalayan syntaxis using different grain-size fractions. Quaternary Science Reviews 124, 124-144 (2015).
11. Fan, X. et al. Earthquake‐Induced Chains of Geologic Hazards: Patterns, Mechanisms, and Impacts. Reviews of Geophysics 57, 421-503 (2019).
12. Fan, X. et al. Spatio-temporal evolution of mass wasting after the 2008 Mw 7.9 Wenchuan earthquake revealed by a detailed multi-temporal inventory. Landslides 15, 2325-2341 (2018).
13. Cui, P., Dang, C., Cheng, Z. & Scott, K. M. Debris Flows Resulting From Glacial-Lake Outburst Floods in Tibet, China. Physical Geography 31, 508-527 (2013).
14. Hu, K., Zhang, X., You, Y., Hu, X., Liu, W. & Li, Y. Landslides and dammed lakes triggered by the 2017 Ms6.9 Milin earthquake in the Tsangpo gorge. Landslides 16, 993-1001 (2019).
15. Brasington, J., Langham, J. & Rumsby, B. Methodological sensitivity of morphometric estimates of coarse fluvial sediment transport. Geomorphology 53, 299-316 (2003).
16. Thompson, C. & Croke, J. Geomorphic effects, flood power, and channel competence of a catastrophic flood in confined and unconfined reaches of the upper Lockyer valley, southeast Queensland, Australia. Geomorphology 197, 156-169 (2013).
17. Wheaton, J. M., Brasington, J., Darby S. E. & Sear, D. A. Accounting for uncertainty in DEMs from repeat topographic surveys: Improved sediment budgets, Earth Surface Process and Landforms 35, 136-156 (2010).
18. Zhuang, Y., Yin, Y., Xing, A. & Jin, K. Combined numerical investigation of the Yigong rock slide-debris avalanche and subsequent dam-break flood propagation in Tibet, China. Landslides 17, 2217-2229 (2020).
19. Lamb, M. P., Finnegan, N. J., Scheingross, J. S. & Sklar, L. S. New insights into the mechanics of fluvial bedrock erosion through flume experiments and theory. Geomorphology 244, 33-55 (2015).
